# Energy Upconversion Using Platinum(II)-BPI Photosensitizers

**DOI:** 10.1021/acs.inorgchem.5c04677

**Published:** 2025-12-10

**Authors:** Ellie N. Payce, Dantong Wang, Jianzhang Zhao, Peter N. Horton, Simon J. Coles, James A. Platts, Simon J. A. Pope

**Affiliations:** † School of Chemistry, Main Building, 2112 Cardiff University, Cardiff CF10 3AT, Cymru/Wales, U.K.; ‡ State Key Laboratory of Fine Chemicals, Frontiers Science Center for Smart Materials, School of Chemical Engineering, 12399 Dalian University of Technology, Dalian 116024, P. R. China; § UK National Crystallographic Service, Chemistry, Faculty of Natural and Environmental Sciences, 7423 University of Southampton, Highfield, Southampton SO17 1BJ, England, U.K.

## Abstract

Five heteroleptic Pt­(II) complexes structurally defined by a diethyl-substituted bis­(2-pyridylimino)­isoindoline (BPI^Et^) core and an ancillary alkynyl coligand are reported. Structural variation across **Pt­(BPI** ^**Et**^ **)­(1–5)** was achieved through different alkyne coligands: phenylacetylene (1), 4-ethynylanisole (2), 3-ethynylthiophene (3), 1-ethynyl-4-fluorobenzene (4), 1-ethynyl-4-dimethylaniline (5). Complexes were fully characterized using a range of spectroscopic and analytical techniques: ^1^H and ^13^C NMR, IR, UV–vis, luminescence, and transient absorption spectroscopies, HRMS, and cyclic voltammetry. Two X-ray structures revealed a subtle deviation from idealized square planar geometry where the Pt atom lies within the plane defined by the three nitrogen donors of BPI^Et^. The redox behavior of the complexes showed one irreversible oxidation between +0.61 and +0.82 V (attributed to Pt^2+/3+^ couple) and two well-defined ligand-based reductions with fully or quasi-reversible character between −1.70 and −2.09 V. Photophysical studies and supporting DFT calculations describe the phosphorescent nature of the complexes (λ_em_ = 625–644 nm in toluene) with strong metal-to-ligand charge transfer (MLCT) character and notable singlet oxygen photogeneration (up to 73%). Triplet–triplet annihilation energy upconversion (TTA-UC) investigations in toluene indicated that this class of triplet emitting complex are viable photosensitizers with an impressive maximum efficiency of Φ_UC_ = 29.6% for **Pt­(BPI** ^**Et**^ **)­(4)**.

## Graphical abstract

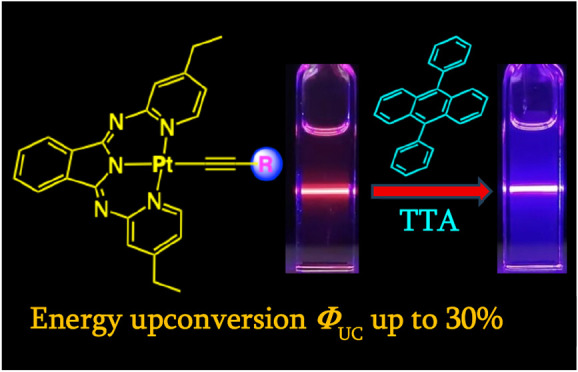

## Introduction

Triplet–triplet annihilation energy upconversion (TTA-UC)^1^ requires both a photosensitizer and an annihilator component and is effective in fluid solution. Important studies have shown that photoactive transition metal complexes can perform well as photosensitizers in TTA-UC; marrying good visible region molar absorptivity and long triplet lifetimes are critical to efficient TTA-UC. Complexes based upon Ru­(II),^2^ Re­(I)^3^ and Ir­(III)^4^ have attracted attention in this context, and generally benefit from the ability to control (and rationally optimize) the photophysical attributes of the complex via ligand design.^5–7^ For example, cyclometalated Ir­(III) complexes can produce world-leading TTA-UC efficiencies (Φ_UC_) up to 39.3% through very subtle iterations of the cyclometalated ligand framework.^8^

Different classes of Pt­(II) complex also have precedent as photosensitizers in TTA-UC, including platinum octaethylporphyrin (**PtOEP**)^9–11^ with a reported TTA-UC efficiency of 23%.^12^ Schiff base [Pt­(N∧O)_2_] complexes have also shown good applicability as photosensitizers,^13^ including chromophore functionalized variants,^14^ as well as organometallic complexes of Pt­(II) with chromophore-functionalized alkynyl ligands inducing extended triplet excited state lifetimes and enabling TTA-UC efficiencies >20%.^15^ In addition, cyclometalated Pt­(II) species of substituted 2-phenylquinoxaline ligands with orange-red phosphorescent character demonstrate ligand-dependent variance in Φ_UC_ = 5.9–14.1% (Figure 1).^16^

**Figure 1:**
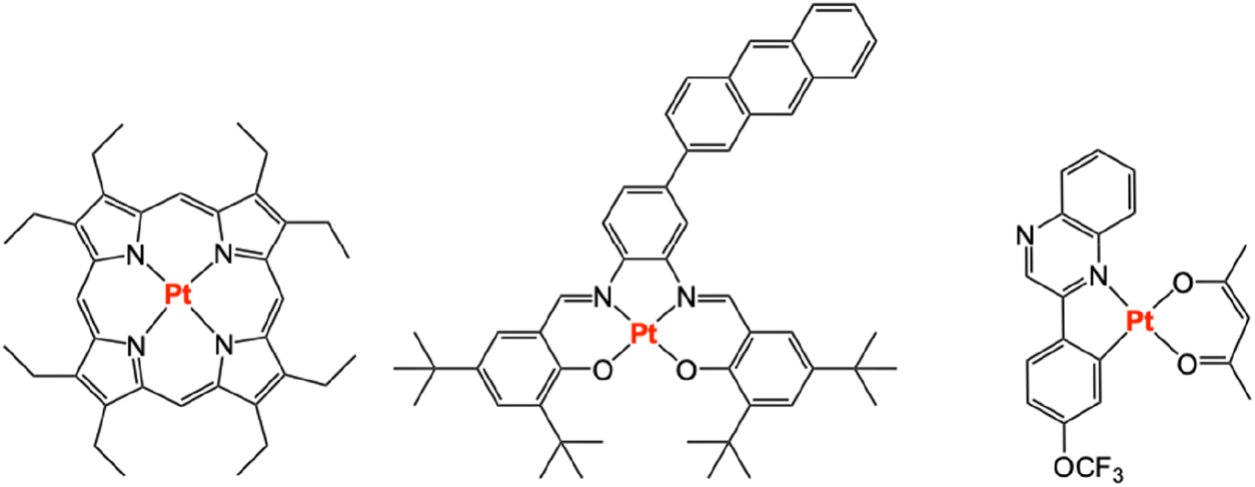
Examples of Pt­(II) complexes successfully utilized as photosensitizers for TTA-UC (left-to-right: porphyrinate, Schiff base and cyclometalated variants).

Pincer type bis­(2-pyridylimino)­isoindoline (BPI) based ligands continue to attract attention in coordination chemistry^17^ with a wide variety of important and potential applications noted,^18^ including as enzymatic mimics.^19^ Although well-suited to a square planar geometry, only a few Pt­(II) complexes of BPI and its derivatives (Figure 2) have been reported over the last 10–15 years. Interestingly, the resultant complexes, [Pt­(BPI)­(L)], can be photoluminescent in the visible region. To tune the optical properties, the BPI ligand architecture (including the site-specific effects of conjugation^20^ and substituents on the isoindolate unit^21^) can be altered^22^ and/or the auxiliary ligand at Pt­(II) can be substituted.^23^ In a further evolution of the BPI framework, unsymmetrical analogues were developed using a stepwise synthetic approach via a key intermediate species (*N*-(pyridin-2-yl)­isoindoline-1,3-diimine). In this manner, different combinations of heterocyclic donor can be incorporated into the pincer structure resulting in tunable excited state properties for the resultant complexes, which absorb strongly in the visible region and are phosphorescent with microsecond lifetimes under deaerated conditions.^24^ The current work builds upon this class of complex and seeks to specifically demonstrate their application as photosensitizers in TTA-UC. To do so, some limitations inherent to the core [Pt­(BPI)­Cl] structures (Figure 2) were addressed, including the relatively poor solubility of the species, and a means to modulate the excited state properties via an ancillary coligand. This Paper describes, via comprehensive structural and spectroscopic characterization, heteroleptic Pt­(II)-BPI type complexes that can be very effective photosensitizers in TTA-UC.

**Figure 2:**
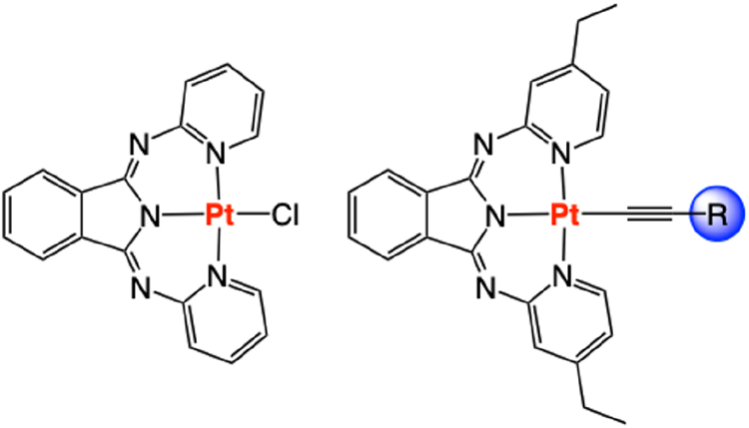
Comparison of the molecular structures of **Pt­(BPI)­Cl** (left) and the complexes developed herein (right).

## Experimental Section

^1^H, ^13^C­{^1^H} NMR spectra were recorded on an NMR-FT Bruker 400 or 500 MHz and spectrometer. ^1^H and ^13^C­{^1^H} NMR chemical shifts (δ) were determined relative to residual solvent peaks with digital locking and are given in ppm. Coupling constants are quoted in Hz. The synthesis of ligand **BPI** ^**Et**^ has been previously reported.^25^ IR spectra were recorded on an ATR equipped Shimadzu IRAffinity-1 spectrophotometer and recorded as solid samples. High-resolution mass spectra were obtained by the staff at Cardiff University. UV–vis studies were performed on a Shimadzu UV-1800 spectrophotometer. Photophysical data were obtained on a JobinYvon–Horiba Fluorolog spectrometer fitted with a JY TBX picosecond photodetection module. The pulsed source was a Nano-LED configured for 295 nm output operating at 1 MHz or 500 kHz. Luminescence lifetime profiles were obtained using the JobinYvon–Horiba FluoroHub single photon counting module and the data fits yielded the lifetime values using the provided DAS6 deconvolution software.

### Cyclic Voltammetry

Cyclic voltammetry was performed using a PalmSens4 potentiostat. Experiments were performed using HPLC grade CH_2_Cl_2_ with an analyte concentration of 1 mM at 293 K, using triply recrystallized [^n^Bu_4_N]­[PF_6_] as the supporting electrolyte at 0.1 M concentration. A three-electrode setup was used, consisting of a platinum disc working electrode, a platinum wire counter-electrode and a silver wire pseudoreference. Solutions were sparged for 10 min with CH_2_Cl_2_ saturated stream of nitrogen gas. Voltammograms were referenced to the ferrocene/ferrocenium redox couple measured using the same conditions.

### Computational Methods

Electronic structure calculations were all performed using density functional theory within the Gaussian09 package.^26^ All calculations were performed using the PBE0 ^27^ functional and def2-SVP basis set, and corresponding effective core potential on platinum.^28^ Full geometry optimizations were performed an all complexes utilizing the self-consistent reaction field model (SCRF) which treats the solvent implicitly as a dielectric continuum. In all cases the solvent chosen was chloroform, the majority of spectroscopic measurements. Minima were confirmed as stationary points through computation of harmonic vibrational frequencies, which showed no imaginary components. These stationary points were used in single point TD-DFT calculations to compute vertical excitation energies using the same basis set and functional. Decomposition of the molecular orbital character was performed using the GaussSum software package.^29^ Orbital plots used the Avogadro package.^30^

### Triplet–Triplet Annihilation Upconversion

A diode pumped solid state (DPSS) laser (473 nm; diameter of the laser spot was ca. 6 mm) was used as the excitation light source for the upconversions. The power of the laser beam was measured with a VLP-2000 pyroelectric power meter. The sample solutions were purged with N_2_ for at least 15 min before measurement. For the upconversion experiments, the mixed solution of the triplet photosensitizers and 9,10-diphenylanthracene (DPA) was degassed for at least 15 min with N_2_. The upconverted fluorescence of DPA was recorded using a RF 5301PC spectrofluorometer. The kinetics of the delayed upconversion luminescence of the TTA upconverion was recorded with the photoluminescence detection mode of the nanosecond transient absorption spectrometer (see later section).

The upconversion quantum yields (Φ_UC_) of all the complexes in toluene were determined with the prompt fluorescence of the Bodipy (Φ_F_ = 0.72 in tetrahydrofuran) as the standard. The upconversion quantum yields were calculated by the equation (shown below), where Φ_UC_, *A*_unk_, *I*_unk_ and η_unk_ represent the quantum yield, absorbance, integrated photoluminescence intensity of the samples (the corresponding parameters with std as subscript are those of the standard compound), and the refractive index of the solvents, respectively. The equation is multiplied by a factor of 2 in order to make the maximum quantum yield to be unity.

$$\Phi_{\mathrm{UC}}=2\Phi_{\mathrm{std}}\left(\frac{A_{\mathrm{std}}}{A_{\mathrm{unk}}}\right)\left(\frac{I_{\mathrm{unk}}}{I_{\mathrm{std}}}\right){\left(\frac{\eta_{\mathrm{unk}}}{\eta_{\mathrm{std}}}\right)}^{2}$$

### Nanosecond Transient Absorption Spectra

The nanosecond transient absorption spectra were recorded on a LP980 laser flash photolysis spectrometer (Edinburgh Instruments, UK). The sample solutions were purged with N2 for 15 min before measurement. The sample solution were excited with nanosecond pulsed laser (Surelite I-10, Continuum Inc.; the wavelength being tunable in the range of 400–2400 nm). The typical laser power is ca. 10 mJ per pulse. The data were processed by L900 software.

### X-ray Crystallography

Single red colored crystals of **Pt­(BPI** ^**Et**^ **)­(2)** (rod shaped, from a mixture of diisopropyl ether and chloroform) and **Pt­(BPI** ^**Et**^ **)­(3)** (plate like, from a mixture of acetonitrile, diisopropyl ether and chloroform) were obtained and data collected following a standard method.^31^ A suitable crystal with dimensions 0.107 × 0.046 × 0.024 mm^3^ for **Pt­(BPI** ^**Et**^ **)­(2)** and 0.111 × 0.093 × 0.021 mm^3^ for **Pt­(BPI** ^**Et**^ **)­(3)** was selected and mounted on a MITIGEN holder in oil on a Rigaku FRE+ diffractometer with Arc)­Sec VHF Varimax confocal mirrors (**Pt­(BPI** ^**Et**^ **)­(2)**) or with HF Varimax confocal mirrors (**Pt­(BPI** ^**Et**^ **)­(3)**), a UG2 goniometer and HyPix 6000HE detector. The crystal was kept at a steady *T* = 100(2) K during data collection. The structure was solved with the 2018/2 version of ShelXT solution program^32^ using dual methods and by using Olex2 1.5 as the graphical interface.^33^ The model was refined with Olex2.refine 1.5.^34^

### Synthesis

#### Synthesis of **Pt­(BPI** ^**Et**^ **)­Cl**

BPI^Et^ (0.153 g, 0.5 mmol) and Pt­(COD)­Cl_2_ (0.165 g, 0.4 mmol) were dissolved in MeOH (12 mL) and heated to reflux for 24 h. The yellow solution rapidly changed color upon addition of DIPEA (81.0 μL, 0.4 mmol) giving a red precipitate. The solid was filtered, washed with MeOH, and dissolved into toluene to afford a red solid (0.176 g, 68%). ^1^H NMR (500 MHz, CDCl_3_) δ_H_ 10.16 (2H, d, *J*_HH_ = 6.7 Hz, CH), 8.09 (2H, dd, *J*_HH_ = 5.5, 3.0 Hz, CH), 7.64 (2H, dd, *J*_HH_ = 5.5, 3.0 Hz, CH), 7.48 (2H, d, *J*_HH_ = 2.4 Hz, CH), 6.90 (2H, dd, *J*_HH_ = 6.7, 2.3 Hz, CH), 2.70 (4H, q, *J*_HH_ = 7.6 Hz, CH_2_), 1.34 (6H, t, *J*_HH_ = 7.6 Hz, CH_3_). ^13^C­{^1^H} NMR (126 MHz, CDCl_3_) δ_C_ 155.8, 151.9, 151.2, 149.8, 137.7, 131.4, 126.2, 122.2, 120.3, 28.0, 13.6. HRMS (ESI) [M + H]^+^ *m*/*z* 585.1133, calculated for [C_22_H_21_N_5_PtCl]^+^, measured 585.1146. IR (ATR, cm^–1^) ν_max_ 419, 465, 480, 698, 750, 781, 824, 833, 882, 895, 920, 1103, 1179, 1204, 1298, 1381, 1410, 1466, 1510, 1580, 1649, 1721, 2872 w, 2928 w, 2961 w, 3123 w. UV–vis (CHCl_3_) λ_max_ (ε, M^–1^cm^–1^): 275 (17475), 345 (9222), 385 (4706), 464 (5807), 490 (6993) nm.

#### Synthesis of **Pt­(BPI** ^**Et**^ **)­(1)**

Pt­(BPI^Et^)Cl (0.040 g, 0.07 mmol) was added to a Schlenk flask, which was then degassed and backfilled with N_2_ four times. Dry DCM (14 mL), phenylacetylene (11.2 μL, 0.10 mmol) and DIPEA (14.3 μL, 0.10 mmol) were added under N_2_. AgBF_4_ (0.020 g, 0.10 mmol) was added in the dark and the solution was stirred overnight at rt. The orange-red solution was filtered through Celite, and the filtrate was concentrated *in vacuo*. The crude product was purified by addition of methanol (15 mL), yielding a precipitate, which was filtered and washed with methanol to afford a red solid (0.033 g, 75%). ^1^H NMR (500 MHz, CDCl_3_) δ_H_ 10.58 (2H, d, *J*_HH_ = 6.6 Hz, CH), 8.04 (2H, dd, J_HH_ = 5.5, 3.0 Hz, CH), 7.62 (2H, dd, *J*_HH_ = 5.5, 3.0 Hz, CH), 7.53–7.50 (2H, m, CH), 7.47 (2H, d, *J*_HH_ = 2.3 Hz, 2H), 7.35–7.28 (2H, m, CH), 7.26–7.21 (1H, m, CH), 6.77 (2H, dd, *J*_HH_ = 6.6, 2.4 Hz, CH), 2.66 (4H, q, *J*_HH_ = 7.6 Hz, CH_2_), 1.32 (6H, t, *J*_HH_ = 7.6 Hz, CH_3_). ^13^C­{^1^H} NMR (126 MHz, CDCl_3_) δ_C_ 156.2, 155.3, 152.8, 150.9, 138.5, 131.6, 131.2, 128.2, 128.0, 127.0, 126.0, 122.2, 120.4, 104.5, 99.8, 28.0, 13.6. HRMS (ESI) [M + H]^+^ *m*/*z* 651.1836, calculated for [C_30_H_26_N_5_Pt]^+^, measured 651.1841 (0.8 ppm). IR (ATR, cm^–1^) ν_max_ 3121 w, 2967 w, 2932 w, 2114 (C≡C), 1576, 1508, 1466, 1383, 1302, 1184, 1158, 1105, 1067, 922, 887, 831, 822, 775, 752, 704, 689, 521; UV–vis (CHCl_3_) λ_max_ (ε, M^–1^cm^–1^): 279 (46,520), 335 (15,670), 347 (19,373), 376 (11,779), 468 (10,742), 490 (10,648) nm.

#### Synthesis of **Pt­(BPI** ^**Et**^ **)­(2)**

The same procedure as for **Pt­(BPI** ^**Et**^ **)­(1)**, except Pt­(BPI^Et^)Cl (0.040 g, 0.07 mmol), 4-ethynylanisole (13.3 μL, 0.10 mmol), DIPA (14.3 μL, 0.10 mmol) and AgBF_4_ (0.020 g, 0.10 mmol) were used. The crude product was purified by addition of methanol (15 mL), yielding a precipitate, which was filtered and washed with methanol to afford a red solid (0.038 g, 82%). ^1^H NMR (500 MHz, CDCl_3_) δ_H_ 10.59 (2H, d, *J*_HH_ = 6.6 Hz, CH), 8.03 (2H, dd, *J*_HH_ = 5.5, 3.0 Hz, CH), 7.60 (2H, dd, *J*_HH_ = 5.5, 3.0 Hz, CH), 7.47–7.42 (4H, m, CH), 6.88–6.84 (2H, m, CH), 6.76 (2H, dd, *J*_HH_ = 6.6, 2.4 Hz, CH), 3.83 (3H, s, CH_3_), 2.65 (4H, q, *J*_HH_ = 7.6 Hz, CH_2_), 1.31 (6H, t, *J*_HH_ = 7.6 Hz, CH_3_). ^13^C­{^1^H} NMR (126 MHz, CDCl_3_) δ_C_ 158.1, 156.2, 155.2, 152.8, 150.8, 138.5, 132.7, 131.1, 126.9, 122.2, 120.5, 120.3, 113.8, 102.0, 99.1, 55.5, 28.0, 13.6. HRMS (ESI) [M + H]^+^ *m*/*z* 681.1942, calculated for [C_31_H_28_N_5_O^195^Pt]^+^, measured 681.1949. IR (ATR, cm^–1^) ν_max_ 3082 w, 3048 w, 2965 w, 2932 w, 2112 (C≡C), 1576, 1503, 1466, 1385, 1304, 1279, 1240, 1209, 1188, 1167, 1105, 1028, 922, 901, 885, 827, 816, 775, 752, 706, 538. UV–vis (CHCl_3_) λ_max_ (ε, M^–1^cm^–1^): 278 (50504), 333 (16834), 348 (19979), 385 (11850), 472 (10798), 489 (10645) nm.

#### Synthesis of **Pt­(BPI** ^**Et**^ **)­(3)**

The same procedure as for **Pt­(BPI** ^**Et**^ **)­(1)**, except Pt­(BPI^Et^)Cl (0.042 g, 0.07 mmol), 3-ethynylthiophene (10.6 μL, 0.11 mmol), DIPA (15.1 μL, 0.11 mmol) and AgBF_4_ (0.021 g, 0.11 mmol) were used. The crude product was purified by flash column chromatography with dichloromethane:petroleum ether (70:30, 100:0) to afford a red solid (0.030 g, 64%). ^1^H NMR (500 MHz, CDCl_3_) δ_H_ 10.54 (2H, d, *J*_HH_ = 6.6 Hz, CH), 8.05 (2H, dd, *J*_HH_ = 5.5, 3.0 Hz, CH), 7.61 (2H, dd, *J*_HH_ = 5.5, 3.0 Hz, CH), 7.47 (2H, dd, *J*_HH_ = 2.4, 0.6 Hz, CH), 7.32 (1H, dd, *J*_HH_ = 3.0, 1.2 Hz, CH), 7.27 (1H, dd, *J*_HH_ = 4.9, 3.1 Hz, CH), 7.20 (1H, dd, *J*_HH_ = 4.9, 1.2 Hz, CH), 6.78 (2H, dd, *J*_HH_ = 6.6, 2.4 Hz, CH), 2.66 (4H, q, *J*_HH_ = 7.6 Hz, CH_2_), 1.32 (6H, t, *J*_HH_ = 7.6 Hz, CH_3_). ^13^C­{^1^H} NMR (126 MHz, CDCl_3_) δ_C_ 156.2, 155.3, 152.8, 150.8, 138.5, 131.2, 130.7, 127.1, 127.0, 125.2, 124.4, 122.2, 120.4, 103.4, 94.1, 28.0, 13.6. HRMS (ESI) [M + H]^+^ *m*/*z* 657.1400, calculated for [C_28_H_24_N_5_S^195^Pt]^+^, measured 657.1432 (4.9 ppm). IR (ATR, cm^–1^) ν_max_ 3067 w, 2963 w, 2928 w, 2118 (C≡C), 1574, 1506, 1464, 1385, 1308, 1184, 1105, 920, 883, 818, 770, 748, 698, 625. UV–vis (CHCl_3_) λ_max_ (ε, M^–1^cm^–1^): 279 (38001), 334 (14427), 347 (17888), 380 (10400), 469 (9589), 490 (9615).

#### Synthesis of **Pt­(BPI** ^**Et**^ **)­(4)**

The same procedure as for **Pt­(BPI** ^**Et**^ **)­(1)**, except Pt­(BPI^Et^)Cl (0.041 g, 0.07 mmol), 1-ethynyl-4-fluorobenzene (12.1 μL, 0.11 mmol), DIPA (14.8 μL, 0.11 mmol) and AgBF_4_ (0.021 g, 0.11 mmol) were used. The crude product was purified by addition of methanol (15 mL), yielding a precipitate, which was filtered and washed with methanol to afford a red solid (0.031 g, 66%). ^1^H NMR (500 MHz, CDCl_3_) δ_H_ 10.49 (2H, d, *J*_HH_ = 6.6 Hz, CH), 8.00 (2H, dd, *J*_HH_ = 5.5, 3.0 Hz, CH), 7.59 (2H, dd, *J*_HH_ = 5.5, 3.0 Hz, CH), 7.49–7.39 (4H, app. m, CH), 7.04–6.96 (2H, app. m, CH), 6.73 (2H, dd, *J*_HH_ = 6.6, 2.4 Hz, CH), 2.64 (4H, q, *J*_HH_ = 7.6 Hz, CH_2_), 1.31 (6H, t, *J*_HH_ = 7.6 Hz, CH_3_). ^13^C­{^1^H} NMR (126 MHz, CDCl_3_) δ_C_ 161.3 (d, ^1^ *J*_CF_ = 246.0 Hz), 156.1, 155.2, 152.7, 150.8, 138.4, 133.0 (d, ^3^ *J*_CF_ = 7.9 Hz), 131.2, 127.0, 124.1, 122.2, 120.3, 115.2 (d, ^2^ *J*_CF_ = 21.7 Hz), 104.0, 98.4, 28.0, 13.6. ^19^F­{^1^H} NMR (377 MHz, CDCl_3_) δ_F_ −114.97 (s, CF). HRMS (ESI) [M + H]^+^ *m*/*z* 669.1742, calculated for [C_30_H_25_N_5_F^195^Pt]^+^, measured 669.1756. IR (ATR, cm^–1^) ν_max_ 3122 w, 3048 w, 2967 w, 2934 w, 2876 w, 2118 (C≡C), 1576, 1508, 1499, 1466, 1383, 1306, 1206, 1186, 1153, 1105, 1093, 1060, 922, 883, 831, 816, 773, 750, 700, 537. UV–vis (CHCl_3_) λ_max_ (ε, M^–1^cm^–1^): 276 (40920), 334 (13551), 347 (16636), 379 (9897), 468 (8790), 492 (8938) nm.

#### Synthesis of **Pt­(BPI** ^**Et**^ **)­(5)**

The same procedure as for **Pt­(BPI** ^**Et**^ **)­(1)**, except Pt­(BPI^Et^)Cl (0.039 g, 0.07 mmol), 1-ethynyl-4-dimethylaniline (0.012 g, 0.08 mmol), DIPA (14.0 μL, 0.10 mmol) and AgBF_4_ (0.020 g, 0.10 mmol) were used. The crude product was purified by flash column chromatography with dichloromethane: methanol (100:0, 95:5) to afford a dark purple solid (0.038 g, 83%). ^1^H NMR (500 MHz, CDCl_3_) δ_H_ 10.70 (2H, d, *J*_HH_ = 6.7 Hz, CH), 8.07 (2H, dd, *J*_HH_ = 5.5, 3.0 Hz, CH), 7.63 (2H, dd, *J*_HH_ = 5.5, 3.0 Hz, CH), 7.50 (2H, d, *J*_HH_ = 2.4 Hz, CH), 7.45–7.41 (2H, m, CH), 6.80 (2H, dd, *J*_HH_ = 6.5, 2.5 Hz, CH), 6.73–6.69 (2H, m, CH), 2.97 (6H, s, CH_3_), 2.67 (4H, q, *J*_HH_ = 7.6 Hz, CH_2_), 1.32 (6H, t, *J*_HH_ = 7.6 Hz, CH_3_). ^13^C­{^1^H} NMR (126 MHz, CDCl_3_) δ_C_ 156.4, 155.3, 152.9, 150.9, 149.0, 138.6, 132.5, 131.2, 126.9, 122.2, 120.4, 116.0, 112.4, 100.2, 99.9, 40.8, 28.0, 13.7. HRMS (ESI) [M + H]^+^ *m*/*z* 694.2258, calculated for [C_32_H_31_N_6_^195^Pt]^+^, measured 694.2254 (−0.6 ppm). IR (ATR, cm^–1^) ν_max_ 3119 w, 3084 w, 2963 w, 2932 w, 2876 w, 2795 w, 2114 (C≡C), 1607, 1574, 1506, 1466, 1427, 1412, 1385, 1350, 1306, 1209, 1184, 1105, 1059, 922, 880, 810, 772, 748, 700, 521. UV–vis (CHCl_3_) λ_max_ (ε, M^–1^cm^–1^): 285 (42,358), 315 (31,734), 330 (27,905), 348 (19,053), 388 (9001), 449 (8080), 460 (8170), 487 (7391), 547 (1851).

## Results and Discussion

### Synthesis of the Complexes

The exploration of Pt­(II) complexes in solution state TTA-UC studies necessitates good solubility of the photosensitizer in solvents compatible with the chosen annihilator, (in this case 9,10-diphenylanthracene, DPA). Prior studies showed that the bis-ethyl-functionalized variant (hereafter abbreviated to as **BPI** ^**Et**^) of BPI (obtained using Siegl’s method^35^ via 4-ethyl-2-aminopyridine) significantly improved the solubility of the resultant neutral complex **Pt­(BPI** ^**Et**^ **)­Cl**. The enhanced solubility characteristics also become advantageous when considering subsequent reactivity of **Pt­(BPI** ^**Et**^ **)­Cl**, which facilitate the substitution reactions of coligands. In this case, the influence of different aromatic alkynes upon the photophysical properties of the complexes was investigated; **Pt­(BPI** ^**Et**^ **)­Cl** was reacted with the relevant alkyne (phenylacetylene (**1**), 4-ethynylanisole (**2**), 3-ethynylthiophene (**3**), 1-ethynyl-4-fluorobenzene (**4**), 1-ethynyl-4-dimethylaniline (**5**)) in the presence of base (Scheme 1). The crude products were isolated as red-colored solids and purified using column chromatography giving a series of neutral, heteroleptic complexes **Pt­(BPI** ^**Et**^ **)­(1–5)** (Scheme 2).

**Scheme 1:**
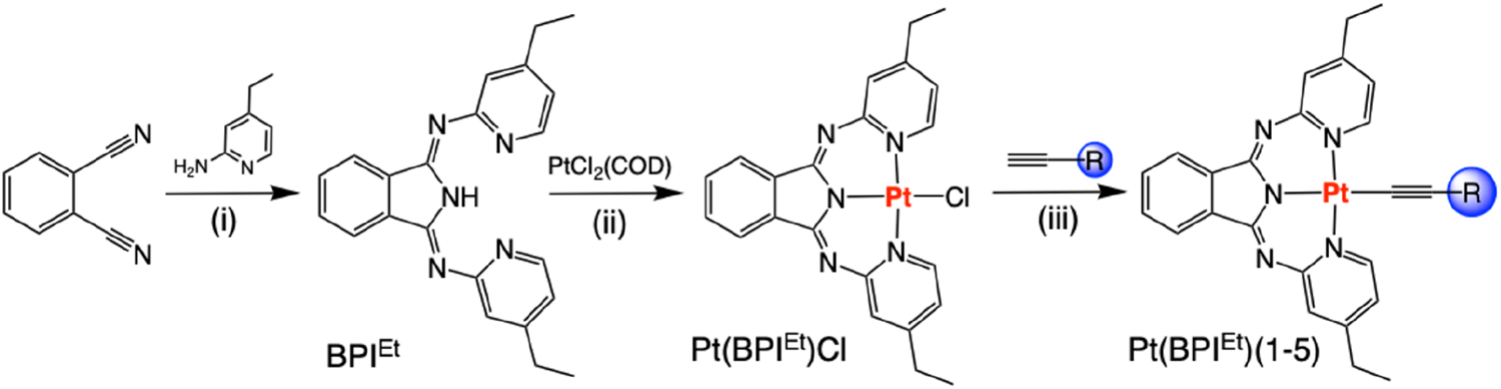
Synthetic route to the heteroleptic Pt­(II) complexes. Reagents: (i) CaCl_2_, *n*-BuOH, reflux; (ii) MeOH, DIPEA, reflux; (iii) DCM, DIPEA, AgBF_4_

**Scheme 2:**
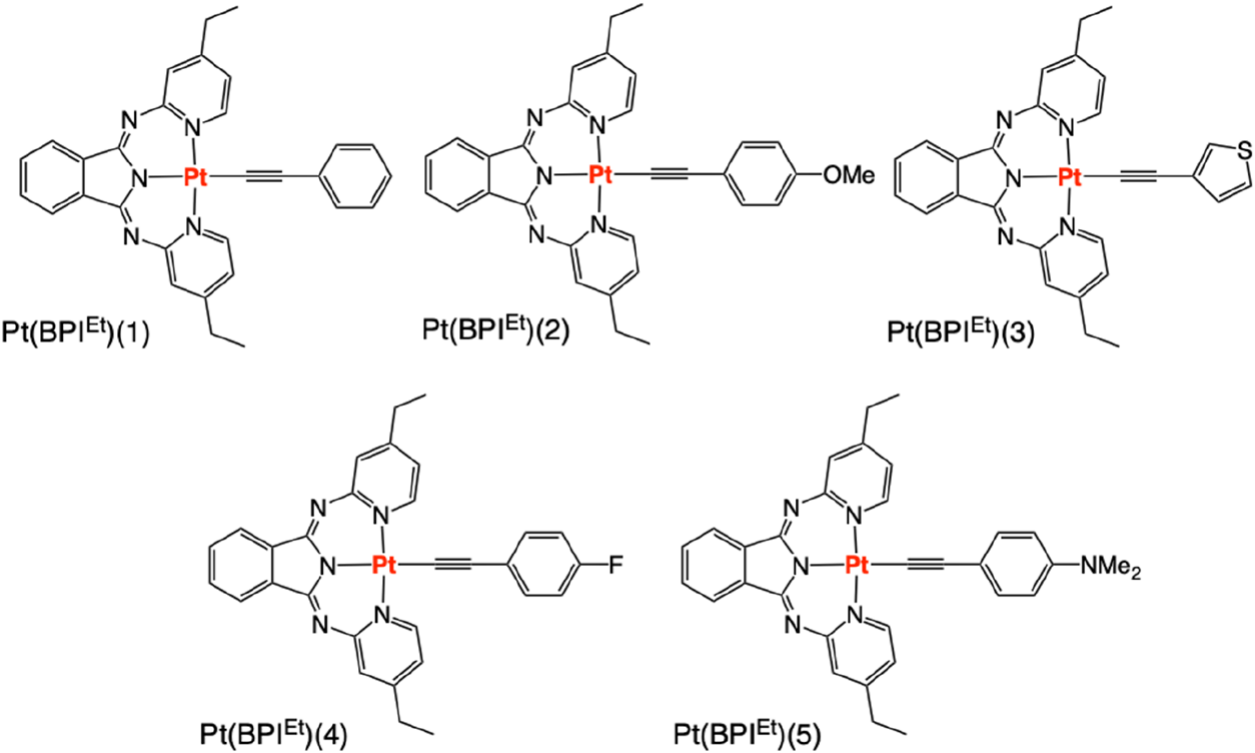
Molecular Structures of the Isolated Pt­(II) Complexes, **Pt­(BPI** ^**Et**^ **)­(1–5)**

As noted in previous studies, square planar Pt­(II) complexes can often demonstrate limiting solubility with a propensity for aggregation. However, this new series of complexes displayed favorable solubility in a range of organic solvents which facilitated comprehensive characterization and analyses. First, ^1^H NMR spectra were obtained for all complexes and showed the retention of the coordinated **BPI** ^**Et**^ ligand, and the addition of resonances associated with the coordinated alkyne (no free alkyne was noted in the spectra). The aromatic resonances within **BPI** ^**Et**^ gave the same pattern as for the precursor **Pt­(BPI** ^**Et**^ **)­Cl**, but the furthest downfield resonance (due to the proton on the C6 position of the pyridinyl donors) was generally observed as a doublet >10.5 ppm, compared to 10.16 ppm for **Pt­(BPI** ^**Et**^ **)­Cl**; within the series, this resonance was most deshielded (δ_H_ 10.70 ppm) for **Pt­(BPI** ^**Et**^ **)­(5)**. In all cases, there was one set of aliphatic signals relating to the ethyl groups within **BPI** ^**Et**^, confirming the symmetrical nature of the complexes. In the case of **Pt­(BPI** ^**Et**^ **)­(5)** an additional singlet at 2.97 ppm was attributed to the dimethylamine fragment of the aniline. In general, coupling to ^195^Pt (*I* = 1/2, 33.8%) was not well-defined in any of the ^1^H NMR spectra, although expansion of the aromatic regions and especially the furthest downfield signals evidence a broadening in the bases of the peaks. The good solubility of the complexes allowed a complete set of ^13^C NMR spectra to be recorded and, again, confirmed the presence of the coordinated ligands.

In particular, the spectra of the complexes presented two alkyne resonances which appeared in the range of 94.1–104.5 ppm (Table 1). The coordinated alkyne carbon is the more upfield of the two signals, as assigned in related alkynyl Au­(III) complexes.^36^ Within the series, the thiophene-functionalized species, **Pt­(BPI** ^**Et**^ **)­(3)**, exhibited the furthest upfield signal (94.1 ppm) for the coordinated alkyne. In the case of **Pt­(BPI** ^**Et**^ **)­(5)** the two alkyne resonances are converged at 99.9 and 100.2 ppm, which presumably reflects the electron donating influence of the *para* substituted dimethylamine group upon the internal alkynyl carbon. In fact, both alkyne carbon resonances shift downfield upon coordination to Pt­(II) (Table 1) with an average Δδ_C_ ca. + 21 ppm. The ^13^C NMR spectrum for **Pt­(BPI** ^**Et**^ **)­(4)** also gave direct evidence for C–F coupling (a doublet at δ_C_ 161.3 ppm with ^1^ *J*_CF_ = 246.0 Hz) which confirmed the presence of the coordinated 1-ethynyl-4-fluorobenzene (further ^2^ *J*_CF_ = 21.7 Hz, δ_C_ = 115.2 ppm, and ^3^ *J*_CF_ = 7.9 Hz, δ_C_ = 133.0 ppm, were identified for the *para*-substituted fluorobenzene moiety). This was further supported by a corresponding ^19^F NMR spectrum which showed a singlet at −114.97 ppm (note fluorobenzene appears at −113.15 ppm). HRMS data was recorded for the neutral complexes and the [M + H]^+^ ion was observed as the dominant cluster, with an isotope pattern consistent with the presence of Pt and fragmentation evidenced via loss of the alkyne to give [Pt­(BPI^Et^)­(MeCN)]^+^; the mass spectrum for **Pt­(BPI** ^**Et**^ **)­(5)** had a [M + 2H]^2+^ cluster at the expected *m*/*z* = 347.6170. Solid state IR spectra of the complexes highlighted the different vibrational modes associated with the ligand constituents. In particular, the coordinated alkyne stretch, which appears in an uncluttered window of the vibrational spectra, was noted as a sharp band at 2112–2118 cm^–1^ (the corresponding free alkyne appear at 2097–2110 cm^–1^). All supporting spectra are reported in SI (Figures S1–S25).

**Table 1:**
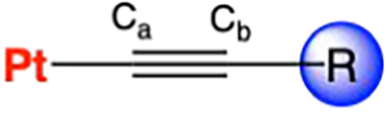
Selected ^13^C NMR Data Showing the Alkyne Chemical Shifts Obtained for the Complexes^a^

	C_a_, δ/ppm	C_b_, δ/ppm	ave. Δδ_C_
**Pt(BPI** ^**Et**^ **)(1)**	99.8 (77.3)	104.5 (83.8)	+21.6
**Pt(BPI** ^**Et**^ **)(2)**	99.1 (75.9)	102.0 (83.8)	+20.7
**Pt(BPI** ^**Et**^ **)(3)**	94.1 (77.1)	103.4 (79.0)	+20.7
**Pt(BPI** ^**Et**^ **)(4)**	98.4 (77.1)	104.0 (82.7)	+21.3
**Pt(BPI** ^**Et**^ **)(5)**	99.9 (74.9)	100.2 (85.0)	+20.1

a Values shown in parentheses are for the corresponding free alkynes.

### X-ray Crystal Structures of Pt­(BPI^Et^)­(**2**) and Pt­(BPI^Et^)­(**3**)

Red single crystals (either rod shaped or plate-like) were successfully isolated for both **Pt­(BPI** ^**Et**^ **)­(2)** and **Pt­(BPI** ^**Et**^ **)­(3)** following slow vapor diffusion of diisopropyl ether into a chloroform solution of the complex. Both structures are monoclinic with **Pt­(BPI** ^**Et**^ **)­(2)** solved in the *C*2/c space group and **Pt­(BPI** ^**Et**^ **)­(3)** solved in the Pc space group (data collection parameters are in Table S1. **Pt­(BPI** ^**Et**^ **)­(3)** reveals some disorder across the thiophene ring and the position of the sulfur atom; **Pt­(BPI** ^**Et**^ **)­(2)** has disorder in the ethyl groups on the pyridinyl donors. Both structures (Figure 3) revealed the expected coordination arrangement for the complexes, with a distorted square planar geometry evidently imposed by the ligands. The Pt atom lies close to the plane formed by the three nitrogen and alkyne carbon donors (0.0115(16) and 0.0019(13) Å, respectively for **Pt­(BPI** ^**Et**^ **)­(2)** and **Pt­(BPI** ^**Et**^ **)­(3)**) which is different to the more distorted structure of **Pt­(BPI** ^**Et**^ **)­Cl**. In both cases the steric requirements of the **BPI** ^**Et**^ ligand are accommodated by a subtle twist of one of the pyridinyl donors, which limits the clash between the H atom in the 6-position of the pyridinyl donors and the ancillary alkyne ligand. In **Pt­(BPI** ^**Et**^ **)­(2)** the anisole ring is clearly twisted (by 87.33(13)°) out of the plane defined by the coordination sphere; for the thiophene analogue the twist is far less (32.37(14)°). Examination of the bond angles, especially the *trans* relationships (N–Pt–N and N–Pt–C) that lie in the range 174.21(14)–178.12(12)°, suggest that **Pt­(BPI** ^**Et**^ **)­(3)** may be slightly closer to idealized square planar geometry. This angular relationship is different to that in **Pt­(BPI)­Cl** where the *trans* N–Pt–N was ca. 170°; replacing the chloride ligand with the alkynyl apparently reduces the distortion within the terdentate BPI ligand. The coordination bond lengths are notable: in **Pt­(BPI** ^**Et**^ **)­(3)** the Pt1–C31 distance is slightly shorter than in **Pt­(BPI** ^**Et**^ **)­(2)**. The Pt–N bond lengths are comparable to previous reports on **Pt­(BPI)­Cl** related complexes and again the Pt1–N3­(indolate) distances are the shortest of the Pt–N bonds (Table 2) within both complexes at 1.992(3) and 2.004(3) Å, respectively. For both complexes, the Pt1–C31–C32 bond angle, noted between 176.7(4)–177.6(3)°, approaches linearity via σ coordination of the aryl acetylide ligands. Examination of the crystallographic packing diagrams (see SI, Figures S26 and S27) revealed the absence of intermolecular Pt·····Pt contacts, but packing is supported by intermolecular BPI^Et^-based π-π interactions for both complexes.

**Figure 3:**
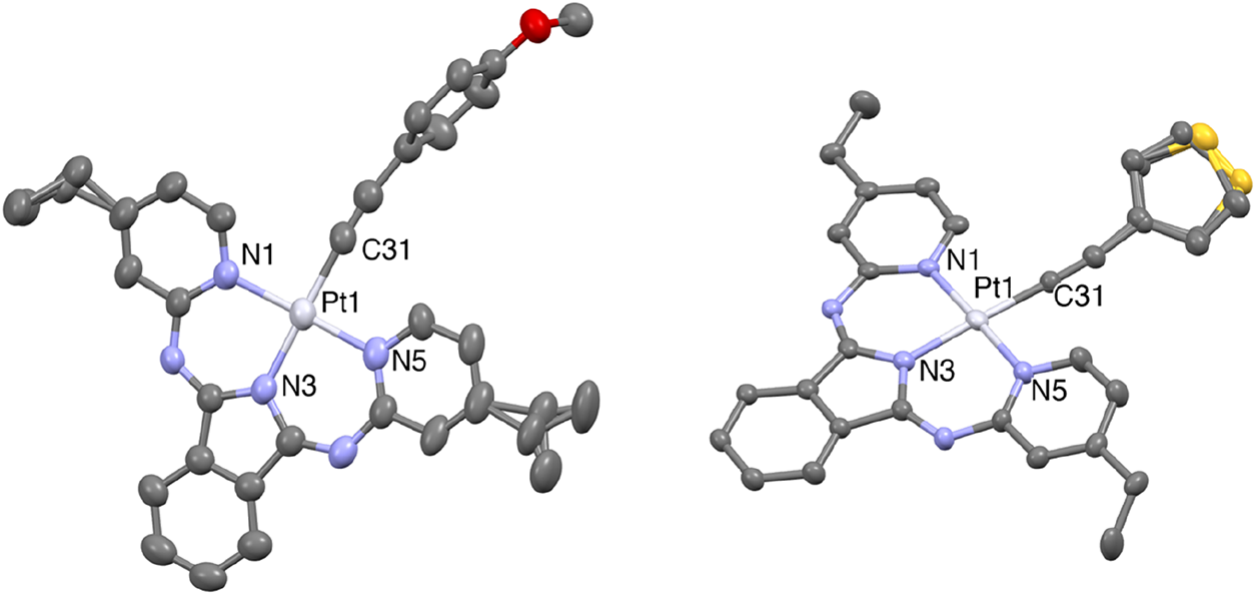
Structural representation obtained from single crystal diffraction studies of **Pt­(BPI** ^**Et**^ **)­(2)** (left) and **Pt­(BPI** ^**Et**^ **)­(3)** (right). Ellipsoids drawn at 50% and hydrogen atoms are omitted.

**Table 2:** Key Structural Parameters That Describe the Coordination Spheres of **Pt­(BPI** ^**Et**^ **)­(2)** and **Pt­(BPI** ^**Et**^ **)­(3)**

bond lengths (Å)					
**Pt(BPI** ^**Et**^ **)(2)**			**Pt(BPI** ^**Et**^ **)(3)**		
Pt1	N1	2.057(3)	Pt1	N1	2.043(3)
Pt1	N3	1.992(3)	Pt1	N3	2.004(3)
Pt1	N5	2.034(3)	Pt1	N5	2.075(3)
Pt1	C31	2.019(4)	Pt1	C31	1.969(4)

bond angles (deg)							
**Pt(BPI** ^**Et**^ **)(2)**				**Pt(BPI** ^**Et**^ **)(3)**			
N3	Pt1	N1	89.36(13)	N3	Pt1	N1	89.44(12)
N5	Pt1	N1	175.51(11)	N5	Pt1	N1	177.40(10)
N5	Pt1	N3	89.76(13)	N5	Pt1	N3	88.61(11)
C31	Pt1	N1	90.34(14)	C31	Pt1	N1	91.69(13)
C31	Pt1	N3	174.21(14)	C31	Pt1	N3	178.12(12)
C31	Pt1	N5	90.98(15)	C31	Pt1	N5	90.31(13)
Pt1	C31	C32	176.7(4)	Pt1	C31	C32	177.6(3)

### Redox properties of the complexes

The electrochemical properties of the complexes were obtained using cyclic voltammetry (1 mM CH_2_Cl_2_ concentration, 0.25 M [^n^Bu_4_N]­[PF_6_] supporting electrolyte and Fc/Fc^+^ as a reference) and the key details are shown in Table 3. First, each complex exhibited an irreversible oxidation process at +0.61 to +0.82 V, which in related compounds has been attributed to a Pt-centered process and thus formation of an unstable Pt­(III) species.^37^ In comparison to the benchmark **Pt­(BPI** ^**Et**^ **)­Cl** (+1.01 V under identical measurement conditions) these alkynyl species appear slightly easier to oxidize, which is consistent with the donating power of the alkyne versus a chloride ancillary ligand. **Pt­(BPI** ^**Et**^ **)­(5)** also gave an additional irreversible oxidation ca. +0.2 V, which is tentatively assigned to a tertiary amine redox process.^38^ Each of the complexes showed two fully reversible waves (Figure 4) within the reduction window with very little variance across the series; in alignment with previous reports for **Pt­(BPI)­Cl** and related complexes, these are attributed to **BPI** ^**Et**^ based processes. The very subtle differences in reduction potentials presumably reflects the modulation in electron density at Pt caused by variance of the alkyne coligand.

**Figure 4:**
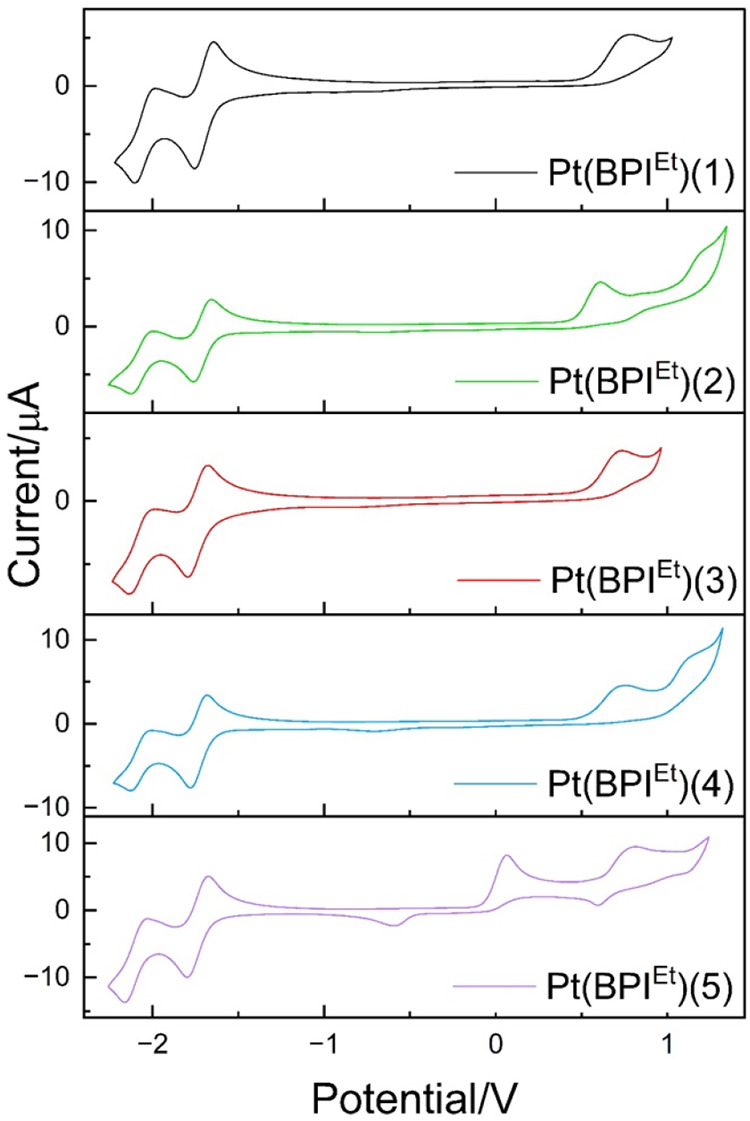
Cyclic voltammograms of **Pt­(BPI** ^**Et**^ **)­(1–5)**. Measurements were recorded in degassed CH_2_Cl_2_, 293 K and 0.25 M [*n*-Bu_4_N]­[PF_6_] at a scan rate of 100 mV/s, and referenced to Fc/Fc^+^.

**Table 3:** Redox Potentials Obtained from the Cyclic Voltammetry of the Series of Pt­(II) Complexes^a^

complex	*E*_ox_	*E*_red1_	*E*_red2_
**Pt(BPI** ^**Et**^ **)(1)**	+0.79	–1.70^b^	–2.04^b^
**Pt(BPI** ^**Et**^ **)(2)**	+0.61	–1.71^b^	–2.07^b^
**Pt(BPI** ^**Et**^ **)(3)**	+0.73	–1.73^b^	–2.07^b^
**Pt(BPI** ^**Et**^ **)(4)**	+0.75	–1.73^b^	–2.07^c^
**Pt(BPI** ^**Et**^ **)(5)**	+0.82	–1.74^b^	–2.09^b^

a Electrochemical potentials reported in Volts (V) relative to Fc/Fc^+^ (0 V). Measurements were recorded in degassed CH_2_Cl_2_, 293 K and 0.25 M [*n*-Bu_4_N]­[PF_6_] at a scan rate of 100 mV/s. All observed oxidation waves were irreversible.

b *E*_1/2_ values from reversible waves.

### Spectroscopic Properties

Experimental data was acquired using a variety of optical spectroscopy techniques; supporting theoretical approaches were also used to provide additional insight on the electronic properties of the complexes. As noted previously, the UV–vis absorption of free **BPI** ^**Et**^ revealed a set of relatively strong absorption bands across the UV and blue edge of the visible region. The peak maxima between 260 and 430 nm are attributed to the various aromatic components that yield intense allowed π → π* transitions. The rigidity of the **BPI** ^**Et**^ structure likely promotes the vibronic structure evident in the lowest energy band envelope. Once coordinated to Pt­(II) the **BPI** ^**Et**^-based transitions are perturbed, and a new visible band at 450–550 nm was observed and attributed to a spin-allowed metal-to-ligand charge transfer (^1^MLCT) transition (Pt­(5d)→L­(π*)).The high intensity (ε > 30000 M^–1^cm^–1^) bands <300 nm include π → π* transitions associated with the ancillary alkynyl ligand; it is noteworthy that **Pt­(BPI** ^**Et**^ **)­(5)** also reveals a broad shoulder absorption ca. 325 nm, which can be attributed to the extended conjugation of the coordinated 1-ethynyl-4-dimethylaniline (Table 4).

**Table 4:** UV–vis Absorption Data for the Pt­(II) Complexes (CHCl_3_, 10^–5^ M)

complex	λ_abs_/nm (ε × 10^4^/ M^–1^cm^–1^)
Pt(BPI^Et^)(1)	279 (4.652), 335 (1.567), 347 (1.937), 376 (1.178), 468 (1.074), 490 (1.065)
Pt(BPI^Et^)(2)	278 (5.050), 333 (1.683), 348 (1.998), 385 (1.185), 472 (1.080), 489 (1.065)
Pt(BPI^Et^)(3)	279 (3.800), 334 (1.443), 347 (1.789), 380 (1.040), 469 (0.959), 490 (0.962)
Pt(BPI^Et^)(4)	276 (4.092), 334 (1.355), 347 (1.664), 379 (0.990), 468 (0.879), 492 (0.894)
Pt(BPI^Et^)(5)	285 (4.236), 315 (3.173), 330 (2.791), 348 (1.905), 388 (0.900), 449 (0.808), 460 (0.817), 487 (0.739), 547 sh (0.185)

The favorable solubility characteristics of the complexes also allowed absorption spectra to be obtained in solvents of varying polarity. Subtle variation in the appearance of the bands was noted, especially the lowest energy MLCT band, where more polar solvents (MeCN, MeOH) induced a hypsochromic shift relative to nonpolar (toluene, *n*-hexane) solvents (Figure 5) implying a possible negative solvatochromic effect.

**Figure 5:**
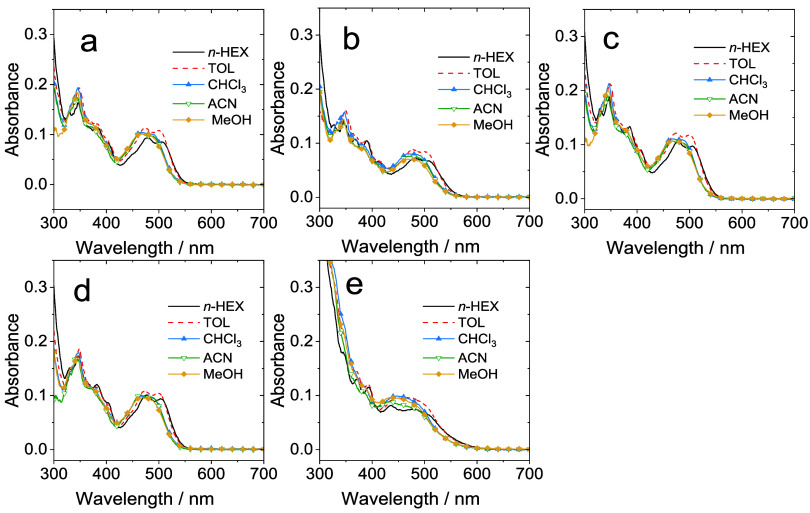
Experimental UV–vis absorption spectra of **Pt­(BPI** ^**Et**^ **)­(1–5)** (a)–(e) in different solvents (10^–5^ M).

Calculations were performed on the Pt­(II) complexes to support the discussion and assignments of the absorption spectra using time dependent DFT (TD-DFT) and molecular orbital decomposition analysis. The calculated optimized geometries for the complexes were in excellent agreement with the experimental structural data obtained **Pt­(BPI** ^**Et**^ **)­(2)** and **Pt­(BPI** ^**Et**^ **)­(3)**; bond lengths, angles and the general coordination sphere were very well replicated by the calculations.

Orbital plots (Figures 6 and S28) indicate that the HOMO is delocalized across Pt (ca. 20%), BPI^Et^ (ca. 20%) and the alkyne (up to 60%), specifically a π-bonding MO. The LUMO is predicted to be localized on the BPI^Et^ ligand, with little or no contribution from Pt or alkyne. In all cases the Pt-BPI^Et^ core is almost exactly planar, but the alkyne ligand lies markedly out of the coordination plane, an effect that is largest in **Pt­(BPI** ^**Et**^ **)­(2)** and **Pt­(BPI** ^**Et**^ **)­(5)**.

**Figure 6:**
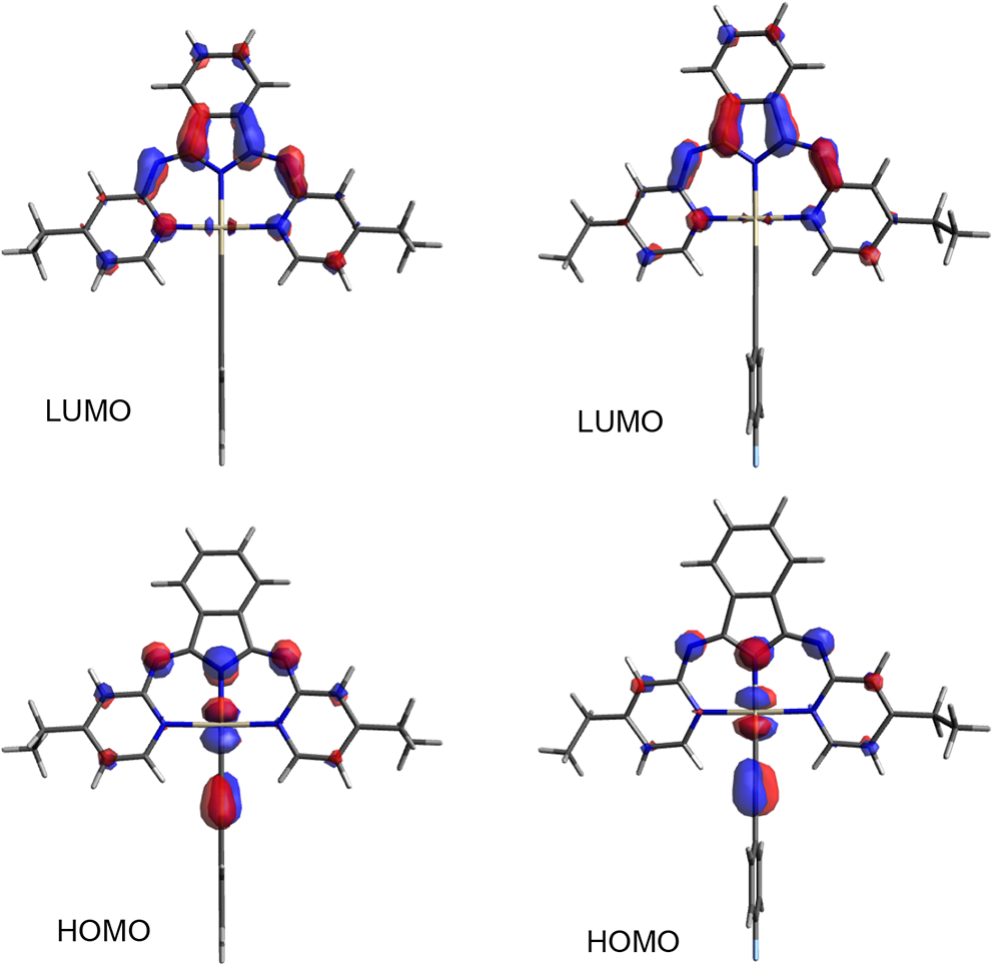
Comparison of the pictorial representation of the frontier orbitals for **Pt­(BPI** ^**Et**^ **)­(1)** (left) and **Pt­(BPI** ^**Et**^ **)­(4)** (right).

In each case, DFT predicted (Table 5) strong absorption around 470–490 nm: in three of the complexes this corresponds to the HOMO → LUMO excitation. The HOMO encompasses Pt, BPI^Et^ and alkyne contributions; the relative contributions vary according to the nature of the alkyne, but overall, this implies an admixture of MLCT, LLCT and ILCT character to the transition. In **Pt­(BPI** ^**Et**^ **)­(2)** and **Pt­(BPI** ^**Et**^ **)­(5)** this strong absorption is predicted to stem from HOMO–1 → LUMO. In addition, for **Pt­(BPI** ^**Et**^ **)­(5)**, and to a lesser extent **Pt­(BPI** ^**Et**^ **)­(2)**, a much weaker, longer wavelength band is predicted from an alkyne-based HOMO to BPI^Et^-based LUMO (implying a LLCT transition). Both of these complexes share the electron donating substituent on the phenylacetylene unit, which may raise the HOMO level. Experimentally, **Pt­(BPI** ^**Et**^ **)­(5)** exhibited a broader tail to the visible absorption band (525–575 nm) which may be attributable to this assignment (furthermore, this band was shown to diminish upon addition of *p*-toluenesulfonic acid which will protonate the NMe_2_ group). Overall, DFT nicely reproduces the important transitions of the absorption spectra of the complexes, particularly the band in the visible region around 500 nm (Figure S29).

**Table 5:** Calculated Energies of the Frontier Orbitals and Predictions of Selected Spectral and Structural Parameters for the Series of Pt­(II) Complexes

complex	HOMO/eV	LUMO/eV	S_0_ → S_n_/nm (f, strength)	C^a^ to Pt(BPI) plane/Å
**Pt(BPI** ^**Et**^ **)(1)**	–6.02	–2.61	470 (0.20, s)	0.616
**Pt(BPI** ^**Et**^ **)(2)**	–5.71, −5.97	–2.57	489 (0.01, w), 472 (0.18, s)	0.838
**Pt(BPI** ^**Et**^ **)(3)**	–5.94	–2.61	475 (0.16, s)	0.651
**Pt(BPI** ^**Et**^ **)(4)**	–6.03	–2.60	467 (0.21, s)	0.690
**Pt(BPI** ^**Et**^ **)(5)**	–5.15, −5.91	–2.51	585 (0.00, w), 476 (0.16, s)	0.847

a Defined as the ipso carbon atom of the ring.

An orbital decomposition analysis predicted the different contributions to the HOMO and LUMO and, as shown (Table 6), the HOMO is characterized by Pt (5d) and ligand-based orbitals from both BPI^Et^ and alkyne moieties. The LUMO is almost exclusively localized on the BPI^Et^ ligand, such that the important HOMO–LUMO transitions are predicted to contain both MLCT and ILCT contributions.

**Table 6:** Partial Densities (%) of Calculated HOMO/HOMO–1 and LUMO for **Pt­(BPI** ^**Et**^ **)­(1–5)** Partitioned Into Three Main Components (Pt, BPI^Et^ and Alkyne)

**Pt(BPI** ^**Et**^ **)(1)**			
orbital	Pt	BPI^Et^	alkyne
LUMO	3	97	0
HOMO	24	55	21

**Pt(BPI** ^**Et**^ **)(2)**			
LUMO	2	98	0
HOMO	6	4	90
HOMO–1	25	54	21

**Pt(BPI** ^**Et**^ **)(3)**			
LUMO	3	97	0
HOMO	14	20	66

**Pt(BPI** ^**Et**^ **)(4)**			
LUMO	2	98	0
HOMO	23	55	22

**Pt(BPI** ^**Et**^ **)(5)**			
LUMO	3	97	0
HOMO	3	2	95
HOMO–1	27	49	25

Additional DFT calculations (using UPBE0/def2-SVP level) were used to generate the isosurfaces of electron spin density of the optimized triplet state geometries of each of the complexes. The pictorial representations of these calculations are shown in Figure 7 and show appreciable spin density on both ligands and the Pt center. In all cases the spin density on Pt is in a *d*_*xz*_ orbital (where the alkyne lies along x, z is perpendicular to coordination plane). Spin density is delocalized over most of the BPI^Et^ ligand including the indolate and pyridine rings, and the bridging N. Within the alkyne, the calculations suggest the triplet state exhibits spin density in the π-orbitals of the alkyne and conjugated ring. In the cases where electron donating groups are attached at the *para* position (**2** and **5**), the spin density extends to a π-orbital on the O or N substituent, whereas in **3** (the thiophene variant) the spin density is much less delocalized, encompassing only a single carbon to any appreciable extent.

**Figure 7:**
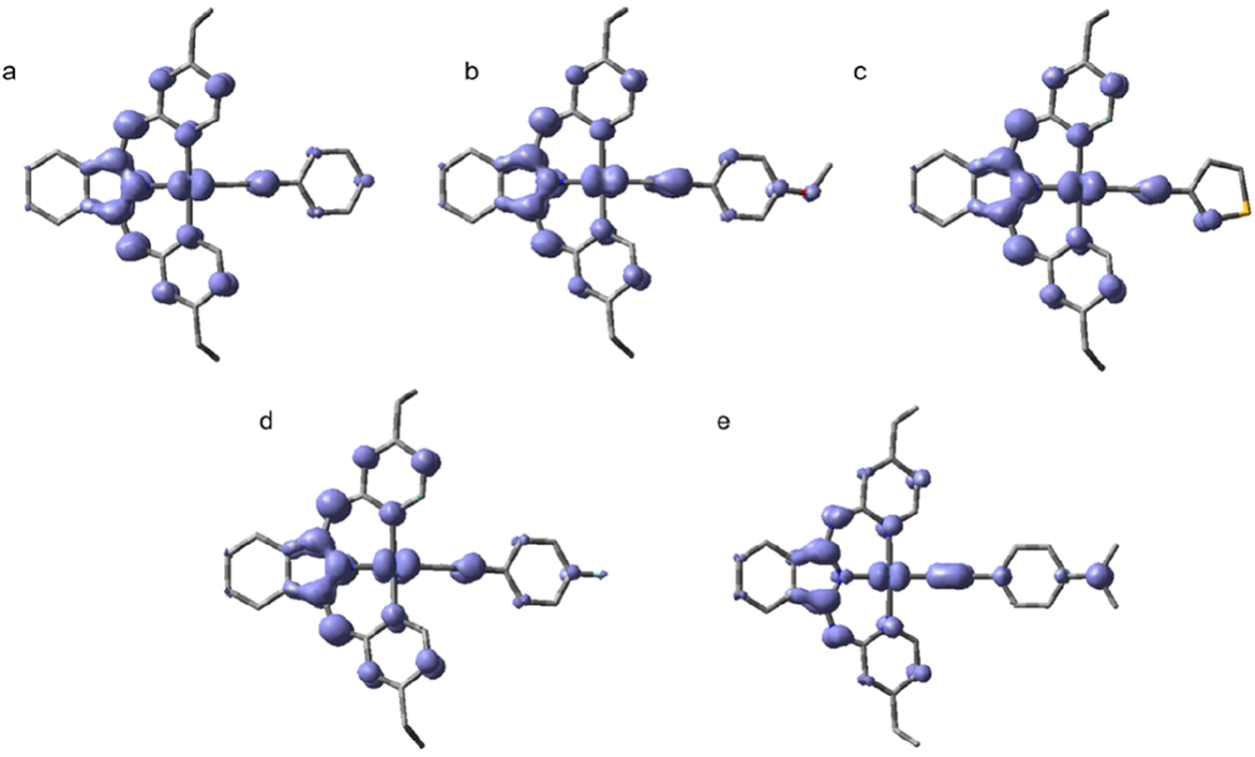
Isosurfaces of spin density at the optimized triplet state geometries of (a) **Pt­(BPI** ^**Et**^ **)­(1)**, (b) **Pt­(BPI** ^**Et**^ **)­(2)**, (c) **Pt­(BPI** ^**Et**^ **)­(3)**, (d) **Pt­(BPI** ^**Et**^ **)­(4)**, (e) **Pt­(BPI** ^**Et**^ **)­(5)** in *n*-hexane. Performed by DFT method at the UPBE0/def2-SVP level with Gaussian 09, plotted at 0.05 au isosurface.

The photophysical measurements on the complexes in toluene showed that each of the species was photoluminescent (following excitation at 475 nm) with emission wavelengths falling within in the range 623–644 nm (Table 6). This implies the ancillary alkyne, as expected from the redox properties, has a subtle influence upon the energy of the emitting state. The effect of solvent (Figure 8; see also Table S3) was varied leading to changes in the appearance and intensity of the emission bands; typically more polar solvents (MeCN, MeOH) led to a quite profound diminution of the emission intensity (both **Pt­(BPI** ^**Et**^ **)­(2)** and **Pt­(BPI** ^**Et**^ **)­(5)** become essentially nonemissive in MeOH), together with a small bathochromic shift. Qualitatively it was noted that **Pt­(BPI** ^**Et**^ **)­(5)** appeared to be weakly emissive.

**Figure 8:**
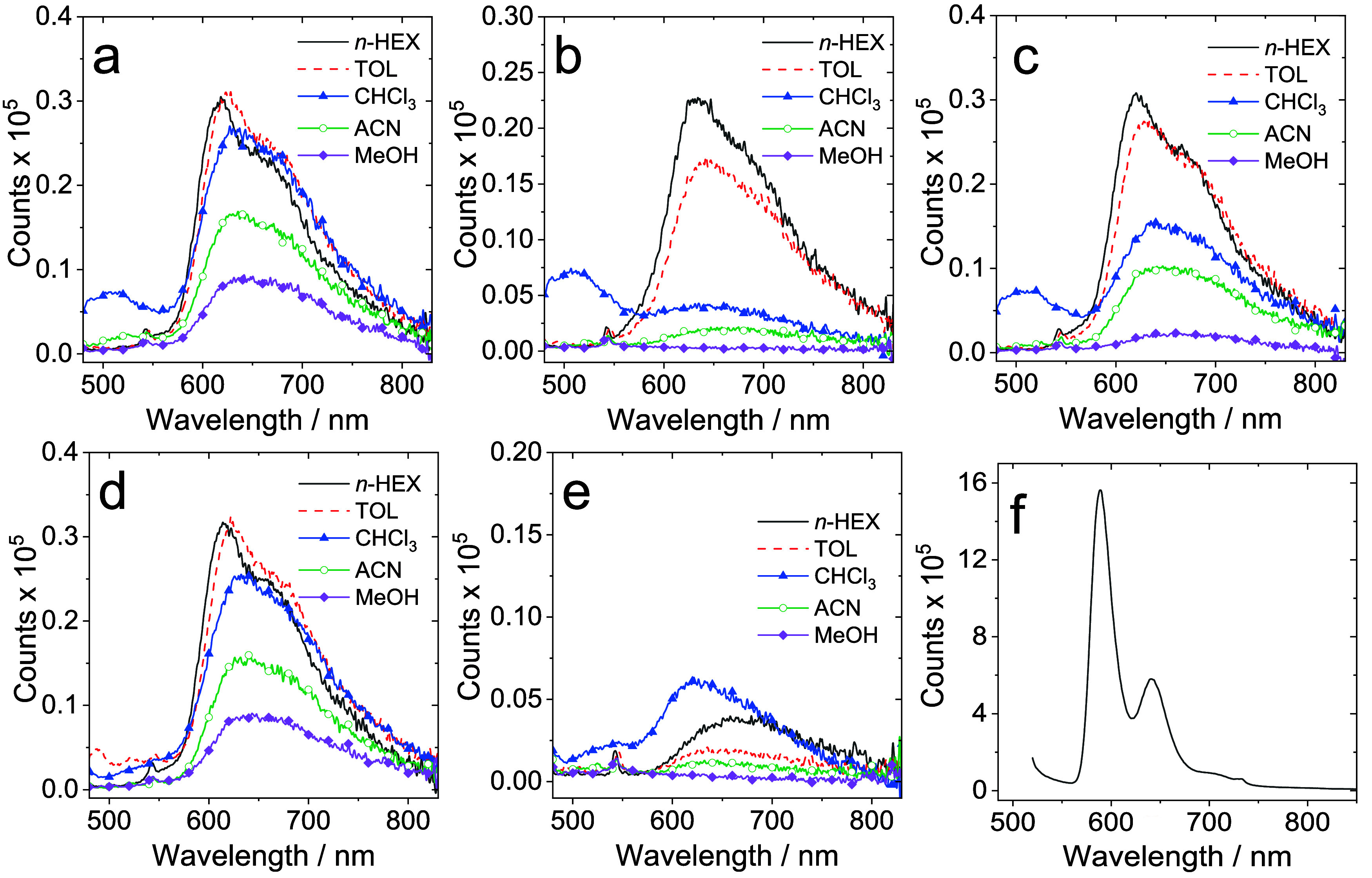
Photoluminescence emission spectra of (a)–(e) **Pt­(BPI** ^**Et**^ **)­(1–5)** in various solvents (293 K, aerated; λ_ex_ = 470 nm); optically matched solutions (*A* = 0.1) were used in each panel; (f) shows **Pt­(BPI** ^**Et**^ **)­(3)** recorded as a 2-MeTHF glass at 77 K (excited with a laser at 510 nm).

Time-resolved measurements in aerated and deoxygenated toluene show the triplet character of the emissive state (Table 7). The lifetimes were typically <300 ns under aerated conditions, which extended to the microsecond domain upon deoxygenation. Comparisons using a variety of different polarity solvents (Table S3) yielded lifetime values which were generally shorter in more polar solvents. **Pt­(BPI** ^**Et**^ **)­(5)** exhibited very pronounced solvent-dependent values often showing a short-lived component to the decay. Furthermore, the triplet nature of the complexes was exemplified through the photogeneration of singlet oxygen, ^1^O_2_ (^1^Δ_g_); quantum yields (Φ_Δ_) are listed in Table 7. In toluene, **Pt­(BPI** ^**Et**^ **)­(1)** generally gave the highest value while **Pt­(BPI** ^**Et**^ **)­(5)** was the least effective. Interestingly, sensitization to ^1^O_2_ was strongly solvent dependent (Table 8). In (nonpolar) toluene and *n*-hexane **Pt­(BPI** ^**Et**^ **)­(1–4)** all gave relatively good performance with yields 21.2–73.0%. In more polar solvents **Pt­(BPI** ^**Et**^ **)­(2)** and **Pt­(BPI** ^**Et**^ **)­(5)** (note these complexes possess -OMe and -NMe_2_ functional groups, respectively) tended to show the lowest ^1^O_2_ yields, with zero values recorded in methanol. In fact, in methanol, where hydrogen bonding plays an important role in molecular diffusion rates, ^1^O_2_ yields were lowest across the series. Taken together the photoluminescence measurements support the assignment of the emitting state having triplet and charge transfer character.

**Table 7:** Photoluminescence Data for the Pt­(II) Complexes in Toluene (10^–5^ M)^a^

complex	λ_em_/nm^b^	τ/μs^c^	degas τ/μs^c^	*Φ*_Δ_ ^d^
**Pt(BPI** ^**Et**^ **)(1)**	625	0.3	1.1, 1.5 (63%)	73.0
**Pt(BPI** ^**Et**^ **)(2)**	644	0.1	0.2 (97%), 1.1	21.2
**Pt(BPI** ^**Et**^ **)(3)**	630	0.2	0.8 (97%), 1.4	36.9
**Pt(BPI** ^**Et**^ **)(4)**	624	0.3	0.9, 1.5 (83%)	31.3
**Pt(BPI** ^**Et**^ **)(5)**	644	0.3	0.6, 1.1 (88%)	3.4

a All measurements obtained at 293 K unless otherwise stated using λ_ex_ = 510 nm;

b Emission maximum;

c Observed lifetime;

d Singlet oxygen quantum yield (Φ_Δ_) in toluene with [Ru­(bipy)_3_[PF_6_]_2_ used as standard compound (Φ_Δ_ = 57% in DCM), λ_ex_ = 480 nm.

**Table 8:** A Comparison of the Solvent Dependent Singlet Oxygen Quantum Yields (%) for the Pt­(II) Complexes^a^

complex	hexane	acetonitrile	methanol	chloroform	toluene	ave.
**Pt(BPI** ^**Et**^ **)(1)**	26.6	35.1	7.1	25.0	73.0	33.4
**Pt(BPI** ^**Et**^ **)(2)**	25.6	5.8	0	4.8	21.2	11.5
**Pt(BPI** ^**Et**^ **)(3)**	26.9	26.5	3.8	16.1	36.9	22.0
**Pt(BPI** ^**Et**^ **)(4)**	31.8	38.5	10.5	23.4	31.3	27.1
**Pt(BPI** ^**Et**^ **)(5)**	5.7	4.4	0	3.7	3.4	3.4

a Singlet oxygen quantum yield (λ_ex_ = 480 nm) with [Ru­(bipy)_3_]­[PF_6_]_2_ was used as a standard (57% in DCM); ranked left-to-right with increasing solvent viscosities.

Low temperature steady state measurements (77 K, 2-MeTHF glass, λ_ex_ = 510 nm) of the complexes exhibited a vibronically structured emission with two dominant peaks at ca. 590 and 650 nm (for example Figure 8f) with little apparent variance between the different species (Figure S30). Given the known MLCT contribution to the emitting level, the well-defined vibronic progression must relate to the rigidity of the **BPI** ^**Et**^ ligand (the π* orbital must dominate the LUMO) that is common to each complex. Supporting lifetime measurements under these conditions (Figure S31) yielded extended values compared to the room temperature measurements. The complexes **Pt­(BPI** ^**Et**^ **)­(1)** (τ = 3.8, 12.1 (97%) μs), **Pt­(BPI** ^**Et**^ **)­(2)** (τ = 5.0, 11.2 (83%) μs), **Pt­(BPI** ^**Et**^ **)­(3)** (τ = 2.6, 11.6 (97%) μs), **Pt­(BPI** ^**Et**^ **)­(4)** (τ = 2.1, 12.1 (97%) μs) share quite similar lifetime parameters with data fits obtained through a biexponential decay yielding two lifetime values; in each case the dominant value is ca. 12 μs suggesting that the nature of the emitting triplet state is comparable. Again, **Pt­(BPI** ^**Et**^ **)­(5)** (τ = 0.9, 7.5 (92%) μs) was an outlier within the series with a significantly shorter phosphorescence lifetime. Taken together, **Pt­(BPI** ^**Et**^ **)­(5)** clearly shows some degree of quenching of the emitting state, which is attributed to competing photoinduced electron transfer from the -NMe_2_ substituent. This was exemplified with an enhancement in emission intensity noted upon addition of *p*-toluenesulfonic acid to a solution of **Pt­(BPI** ^**Et**^ **)­(5)**. The related complex **Pt­(terpy)­(5)** (where terpy = 2,2′:6′,2″-terpyridine) is nonemissive and shows pronounced pH response.^39^

Time-resolved transient absorption (TA) spectra for the five complexes were recorded in deaerated *n*-hexane. The spectra for **Pt­(BPI** ^**Et**^ **)­(1–4)** are broadly comparable showing three positive bands ca. 400, 550, and 750 nm and a bleach ca. 350 nm; the appearance of these absorptions subtly vary as a function of the structural changes to the alkynyl ligands (Figure 9). This tallies with the DFT predictions that the triplet state electron density distribution includes the alkyne coligand. The excited state absorption (ESA) bands possess decay kinetics that are in the microsecond domain and are likely triplet-to-triplet transitions (T_1_ → T_*n*>1_). The lifetimes are comparable to those obtained from the photoluminescent measurements and suggests that the triplet excited state character noted in the TA spectra probably also relates to the triplet level(s) responsible for the emission of the complexes. In contrast, for **Pt­(BPI** ^**Et**^ **)­(5)** the TA spectrum is very different in appearance with a bleach ca. 500 nm and only very weakly induced ESA (Figure S32).

**Figure 9:**
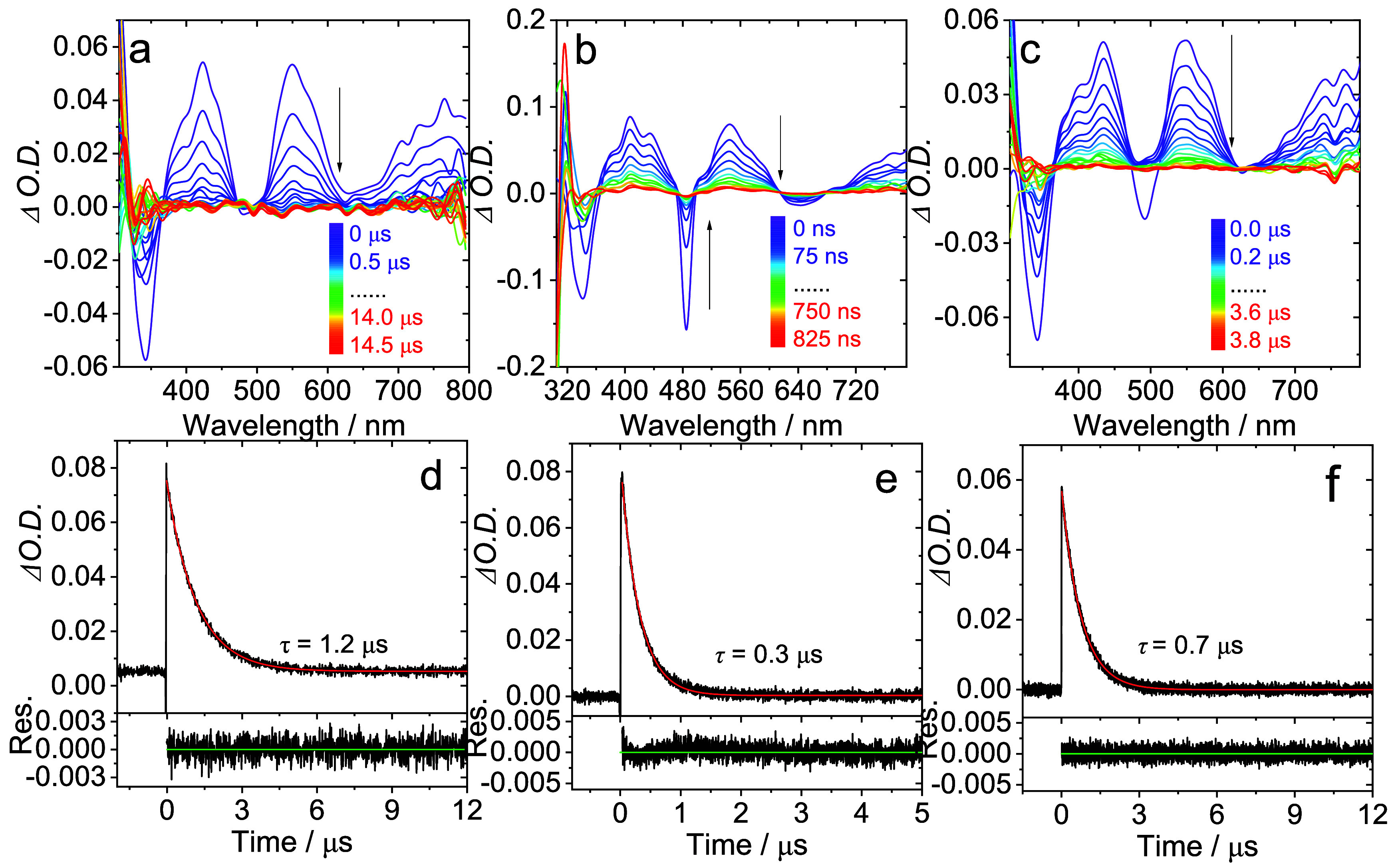
Examples of Nanosecond transient absorption spectra of (a) **Pt­(BPI** ^**Et**^ **)­(1)**, *c* = 3.5 × 10^–5^ M, (b) **Pt­(BPI** ^**Et**^ **)­(2)**, *c* = 8.6 × 10^–5^ M, (c) **Pt­(BPI** ^**Et**^ **)­(3)**, *c* = 3.6 × 10^–5^ M excited at 480 nm in deaerated *n*-hexane. The decay traces of (d) **Pt­(BPI** ^**Et**^ **)­(1)**, *c* = 3.5 × 10^–5^ M, (e) **Pt­(BPI** ^**Et**^ **)­(2)**, *c* = 8.6 × 10^–5^ M, (f) **Pt­(BPI** ^**Et**^ **)­(3)**, *c* = 3.6 × 10^–5^ M at 480 nm in deaerated *n*-hexane.

### Triplet–Triplet Annihilation Energy Upconversion Studies

Solution state TTA-UC studies were undertaken, employing 9,10-diphenylanthracene (DPA) as the annihilator component, and each of the Pt­(II) alkynyl complexes as the photosensitizer. TTA-UC emission spectra were collected for each complex (typically 2.0 × 10^–5^ M) in the presence of DPA (2.0 × 10^–4^ M) in deaerated toluene at room temperature (Figure 10). Laser excitation at 473 nm is selective for the visible absorption band of the photosensitizer Pt­(II) complexes and circumvents direct excitation of the DPA. Importantly, the T_1_ level of DPA is ca. 14,300 cm^–1^ and therefore lies below the triplet emitting levels (the onset of the 77K emission spectra is ca. 17,550 cm^–1^) of all the complexes herein. Therefore, any delayed fluorescence from DPA in the 400–500 nm range can be solely attributed to an upconversion process via TTA. As shown in Figure 10, each complex was a viable photosensitizer for TTA-UC although the delayed fluorescence signal for **Pt­(BPI** ^**Et**^ **)­(5)** was evidently weaker. The TTA-UC performance was quantified (Table 9) regarding upconversion quantum yields (Φ_UC_) which vary from 0.7–29.6% for the series of complexes; **Pt­(BPI** ^**Et**^ **)­(4)** was the best performing, while **Pt­(BPI** ^**Et**^ **)­(5)** produced the weakest upconversion. Furthermore, Figure 11 details the relevant time-resolved spectral data for **Pt­(BPI** ^**Et**^ **)­(4)** where the photosensitizer’s visible emission at 615 nm (τ = 1.4 μs) is partially quenched in the presence of DPA resulting in a reduced phosphorescence lifetime of 0.8 μs. This value correlates directly with the rise-time of the DPA delayed fluorescence measured at 430 nm, which then decays at a rate that yields a lifetime of 84.3 μs. Similar behavior was noted for **Pt­(BPI** ^**Et**^ **)­(1)** which was the second-best performing photosensitizer within the series (Figure S33). This observation marries with the photogenerated singlet oxygen yields, where **Pt­(BPI** ^**Et**^ **)­(1)** and **Pt­(BPI** ^**Et**^ **)­(4)** were the best photosensitizers (see also Figures S33–S36) within the series of complexes. Qualitatively, digital photographs of the laser irradiated cuvettes capture the visual consequence of the energy upconversion process: the red emission of the complexes is attenuated and augmented to varying degrees in the presence of the DPA (Figure 10).

**Figure 10:**
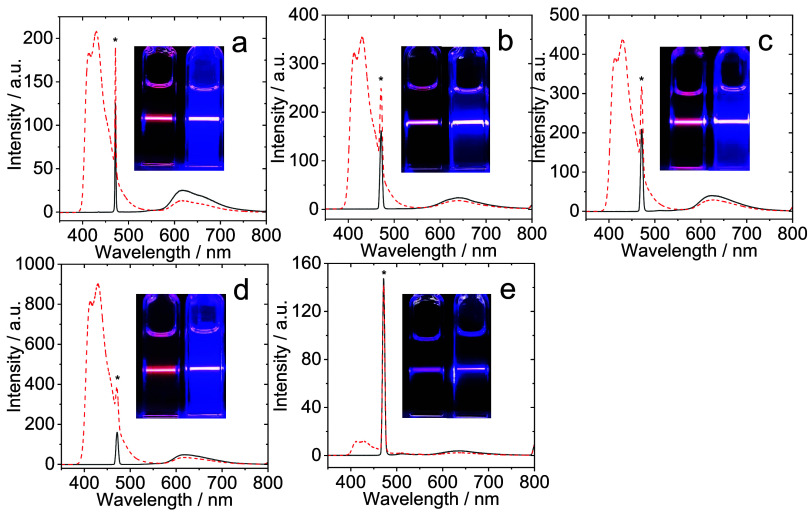
Triplet–triplet annihilation upconversion emission spectra comparing free complex photosensitizer (—) and complex + DPA (red dashed lines). (a) **Pt­(BPI** ^**Et**^ **)­(1)**, *c* = 1 × 10^–5^ M, (b) **Pt­(BPI** ^**Et**^ **)­(2)**, *c* = 2 × 10^–5^ M, (c) **Pt­(BPI** ^**Et**^ **)­(3)**, *c* = 1 × 10^–5^ M, (d) **Pt­(BPI** ^**Et**^ **)­(4)**, *c* = 1 × 10^–5^ M and (e) **Pt­(BPI** ^**Et**^ **)­(5)**, *c* = 2 × 10^–5^ M in deaerated toluene where DPA was used as the annihilator. Excitation was achieved with a continuous laser at λ = 473 nm (power of 6.4 mW for **Pt­(BPI** ^**Et**^ **)­(1)**, power of 5.2 mW for other photosensitizers) under a deaerated toluene atmosphere. *c*(DPA) = 2 × 10^–4^ M, 20 °C. The asterisks indicate the scattered laser signal. The associated photographs show the relevant free complex photosensitizer (left) and complex photosensitizer + DPA (right) in each case.

**Figure 11:**
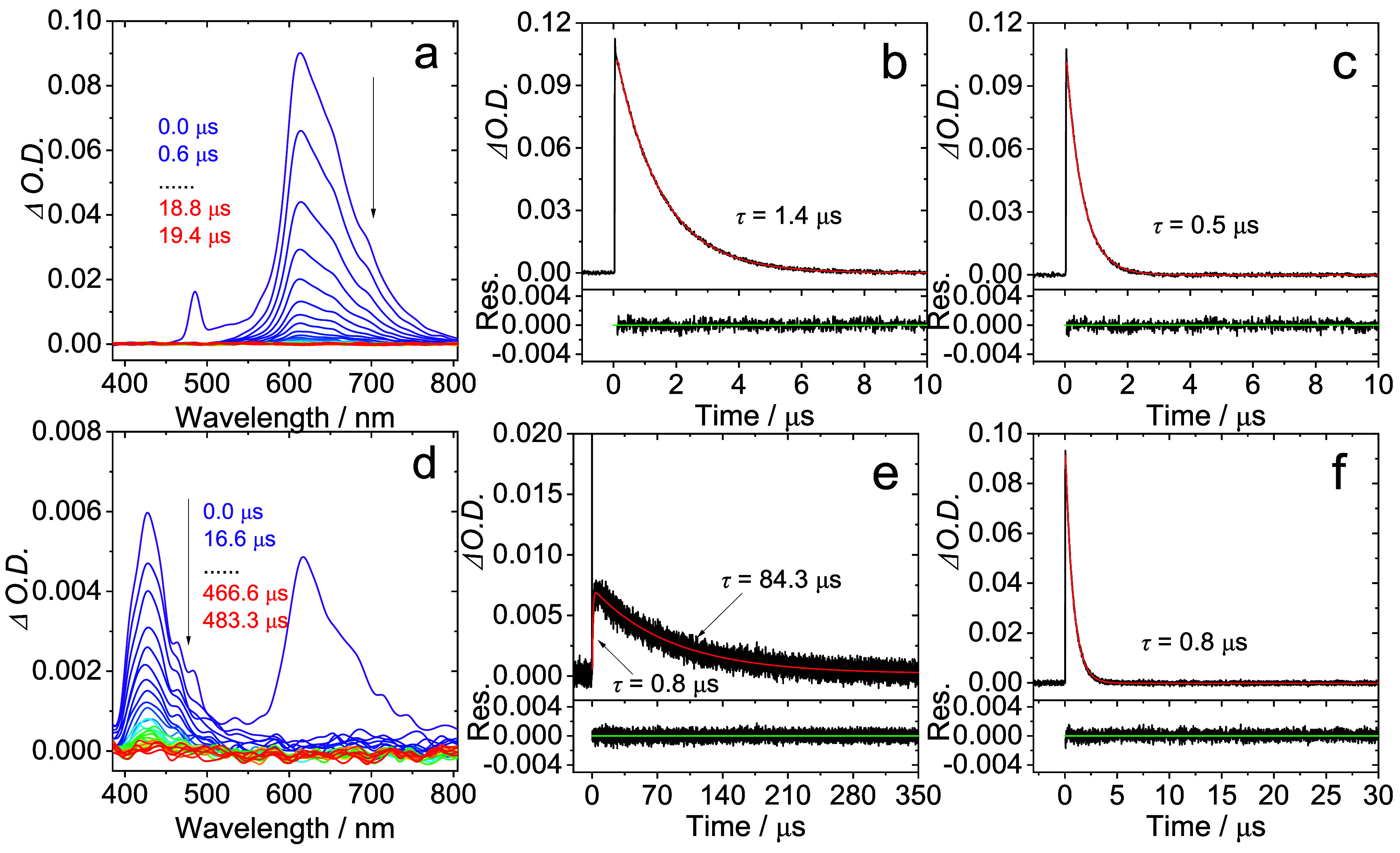
(a) Time-resolved luminescence of **Pt­(BPI** ^**Et**^ **)­(4)** (*c* = 1 × 10^–5^ M); (b) the decay traces of phosphorescence in N_2_; (c) the decay traces of phosphorescence in air; (d) delayed fluorescence with **Pt­(BPI** ^**Et**^ **)­(4)** (*c* = 1 × 10^–5^ M) as the triplet photosensitizer and DPA (*c* = 2.0 × 10^–4^ M) as the triplet acceptor; (e) the decay traces of the emission at 430 nm (^1^DPA* → S_0_); (f) the decay traces of the emission at 615 nm (T_1_ → S_0_); the spike in the delayed fluorescence traces is the scattered laser. Excited with nanosecond pulsed laser at 480 nm. In deaerated toluene, 20 °C.

**Table 9:** TTA-UC Quantum Yields (*Φ*_UC_) Using the Photosensitizer Pt­(II) Complexes and DPA as the Triplet Acceptor

	**Pt(BPI** ^**Et**^ **)(1)** ^a^	**Pt(BPI** ^**Et**^ **)(2)** ^b^	**Pt(BPI** ^**Et**^ **)(3)** ^b^	**Pt(BPI** ^**Et**^ **)(4)** ^b^	**Pt(BPI** ^**Et**^ **)(5)** ^b^
Φ_UC_ (%)^c^	16.8	3.9	10.0	29.6	0.7

a Excited with 473 nm laser (6.4 mW), with the prompt fluorescence of BDP as the inner standard (Φ = 72% in THF).

b Excited with 473 nm laser (5.2 mW), with the prompt fluorescence of BDP as the inner standard (Φ = 72% in THF).

c Upconversion quantum yield (Φ_UC_) using DPA = 2.0 × 10^–4^ M.

Therefore, the best performing complex in this series produces a TTA-UC efficiency which are competitive with the very best Pt­(II) photosensitizers, including platinum octaethylporphyrin. It is evident for this class of Pt­(II)-BPI complex that very subtle alterations of the alkynyl coligands can induce changes to the triplet state energy level, and perhaps more importantly, also influence the excited state decay kinetics.

## Conclusions

In this study, redox active, mixed-ligand Pt­(II) complexes encompassing a bis-ethyl functionalized BPI and an aromatic alkyne coligand, were structurally and spectroscopically characterized. Each of the complexes were photoluminescent at (for example, 625–644 nm in toluene) following excitation into a visible absorption band that is likely to possess strong MLCT character, as supported by computational studies. The complexes are triplet emitters with lifetimes that can extend to the microsecond domain in deoxygenated solvent. Variations in emission wavelength and lifetime were noted across the series, showing that the use of monosubstituted phenylacetylene coligands (where R = H, OMe, F and NMe_2_) can modulate the excited state behavior. The triplet character was further established through a series of studies that described singlet oxygen photogeneration efficiencies, time-resolved transient absorption spectra and, finally, as photosensitizers for triplet–triplet annihilation energy upconversion using DPA as the annihilator. A clear trend emerges where the closely related complexes **Pt­(BPI** ^**Et**^ **)­(1)** and **Pt­(BPI** ^**Et**^ **)­(4)** promote the best photosensitized TTA-UC performances. These complexes share very similar photophysical attributes and are predicted to have very similar HOMO and LUMO characteristics. On the other hand, **Pt­(BPI** ^**Et**^ **)­(2)** and **Pt­(BPI** ^**Et**^ **)­(5)** share electron donating substituents that are predicted to introduce LLCT states that may provide a quenching pathway for the emission. Correspondingly, these complexes show the lowest TTA-UC efficiency values showing that for this class of Pt­(II) complex the alkynyl coligand is critical in determining the overall photosensitizer performance.

## Supplementary Material



## Data Availability

Information on the data underpinning this publication, including access details, can be found in the Cardiff University Research Data Repository at 10.17035/cardiff.30831068.

## Notes

NMR, IR, HRMS spectra for all compounds; data collection parameters for the X-ray crystal structures and packing diagrams; additional photophysical data; additional DFT data (PDF)
