# Supplementary material for: Energy Upconversion Using Platinum(II)-BPI Photosensitizers
Source: Inorg Chem. 2025 Dec 10;64(50):24851–64. doi: 10.1021/acs.inorgchem.5c04677 (PMC12728933; doi:10.1021/acs.inorgchem.5c04677)
Supplement: Supplementary file 1 [file ic5c04677_si_001.pdf]

Supplementary Information

For

Energy Upconversion Using Platinum(II)-BPI Photosensitizers

Ellie N. Payce,[a] Dantong Wang,[b] Jianzhang Zhao,[b] Peter N. Horton,[c] Simon J. Coles,[c] James A. Platts[a] and Simon J. A. Pope[a]*

[a]School of Chemistry, Main Building, Cardiff University, Cardiff CF10 3AT, Cymru/Wales; [b] State Key Laboratory of Fine Chemicals, Frontiers Science Center for Smart Materials, School of Chemical Engineering, Dalian University of Technology, Dalian 116024, P.R. China;[c] UK National Crystallographic Service, Chemistry, Faculty of Natural and Environmental Sciences, University of Southampton, Highfield, Southampton, SO17 1BJ, England, UK.

Email: popesj@cardiff.ac.uk

Contents

Figure S1	^{1}H NMR spectrum of **Pt(BPI^{Et})Cl** in $CDCl_3$ (500 MHz). Inset shows aromatic region.	S4
Figure S2	^{13}C NMR spectrum of **Pt(BPI^{Et})Cl** in $CDCl_3$ (125 MHz).	S4
Figure S3	HRMS spectrum of **Pt(BPI^{Et})Cl**.	S5
Figure S4	Infrared spectrum of **Pt(BPI^{Et})Cl**.	S5
Figure S5	^{1}H NMR spectrum of **Pt(BPI^{Et})(1)** in $CDCl_3$ (500 MHz). Inset shows aromatic region.	S6
Figure S6	^{13}C NMR spectrum of **Pt(BPI^{Et})(1)** in $CDCl_3$ (125 MHz).	S6
Figure S7	HRMS spectrum of **Pt(BPI^{Et})(1)**.	S7
Figure S8	Infrared spectrum of **Pt(BPI^{Et})(1)**.	S7
Figure S9	^{1}H NMR spectrum of **Pt(BPI^{Et})(2)** in $CDCl_3$ (500 MHz). Inset shows aromatic region.	S8
Figure S10	^{13}C NMR spectrum of **Pt(BPI^{Et})(2)** in $CDCl_3$ (125 MHz).	S8
Figure S11	HRMS spectrum of **Pt(BPI^{Et})(2)**	S9
Figure S12	Infrared spectrum of **Pt(BPI^{Et})(2)**.	S9
Figure S13	^{1}H NMR spectrum of **Pt(BPI^{Et})(3)** in $CDCl_3$ (500 MHz). Inset shows aromatic region.	S10
Figure S14	^{13}C NMR spectrum of **Pt(BPI^{Et})(3)** in $CDCl_3$ (125 MHz).	S10
Figure S15	HRMS spectrum of **Pt(BPI^{Et})(3)**.	S11
Figure S16	Infrared spectrum of **Pt(BPI^{Et})(3)**.	S11
Figure S17	^{1}H NMR spectrum of **Pt(BPI^{Et})(4)** in $CDCl_3$ (500 MHz). Inset shows aromatic region.	S12
Figure S18	^{13}C NMR spectrum of **Pt(BPI^{Et})(4)** in $CDCl_3$ (125 MHz).	S12
Figure S19	^{19}F NMR spectrum of **Pt(BPI^{Et})(4)** in $CDCl_3$ (377 MHz).	S13
Figure S20	HRMS spectrum of **Pt(BPI^{Et})(4)**.	S13
Figure S21	Infrared spectrum of **Pt(BPI^{Et})(4)**.	S14
Figure S22	^{1}H NMR spectrum of **Pt(BPI^{Et})(5)** in $CDCl_3$ (500 MHz).	S14
Figure S23	^{13}C NMR spectrum of **Pt(BPI^{Et})(5)** in $CDCl_3$ (125 MHz).	S15
Figure S24	HRMS spectrum of **Pt(BPI^{Et})(5)**.	S15
Figure S25	Infrared spectrum of **Pt(BPI^{Et})(5)**.	S16
Figure S26	Packing diagrams obtained from the X-ray structure of Pt(**BPI^{Et}**)(2).	S18
Figure S27	Packing diagrams obtained from the X-ray structure of **Pt(BPI^{Et})(3)**	S18
Figure S28	Comparison of the pictorial representation of the frontier orbitals for **Pt(BPI^{Et})(2)** (left) and **Pt(BPI^{Et})(3)** (center) and **Pt(BPI^{Et})(3)** (right)	S20
Figure S29	Examples of calculated absorption spectra.	S21
Figure S30	Low temperature photoluminescence emission spectra of selected complexes in frozen 2-MeTHF glass (77 K)	S22
Figure S31	Low temperature lifetime spectra of the complexes in frozen 2-MeTHF glass (77 K)	S22
Figure S32	Nanosecond transient absorption spectra of (a) **Pt(BPI^{Et})(4)** and **Pt(BPI^{Et})(5)**	S23
Figure S33	Time-resolved upconversion luminescence spectra of **Pt(BPI^{Et})(1)**	S23
Figure S34	Time-resolved upconversion luminescence spectra of **Pt(BPI^{Et})(2)**	S24
Figure S35	Time-resolved upconversion luminescence spectra of **Pt(BPI^{Et})(3)**	S24
Figure S36	Time-resolved upconversion luminescence spectra of **Pt(BPI^{Et})(5)**	S25
Table S1	Data collection parameters for the X-ray crystal structures	S17
Table S2	Comparison of experimental (X-ray) and calculated (DFT; values in italics) parameters that describe the coordination geometries	S19

Table S3	Photoluminescence data for the Pt(II) complexes in various solvents	S21
Table S4	Optimized cartesian coordinates	S25

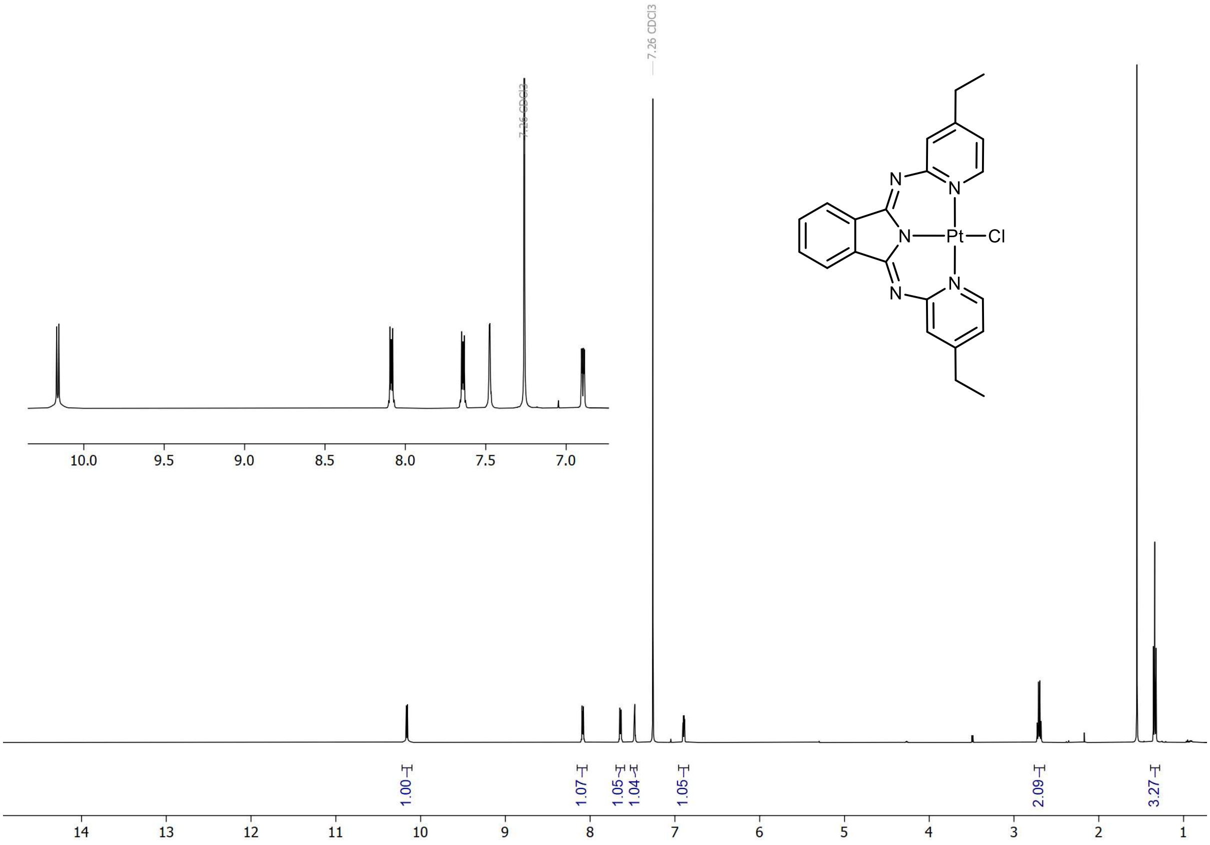

Figure S1. ^{1}H NMR spectrum of **Pt(BPI^{Et})Cl** in $CDCl_3$ (500 MHz). Expansion shows aromatic region.

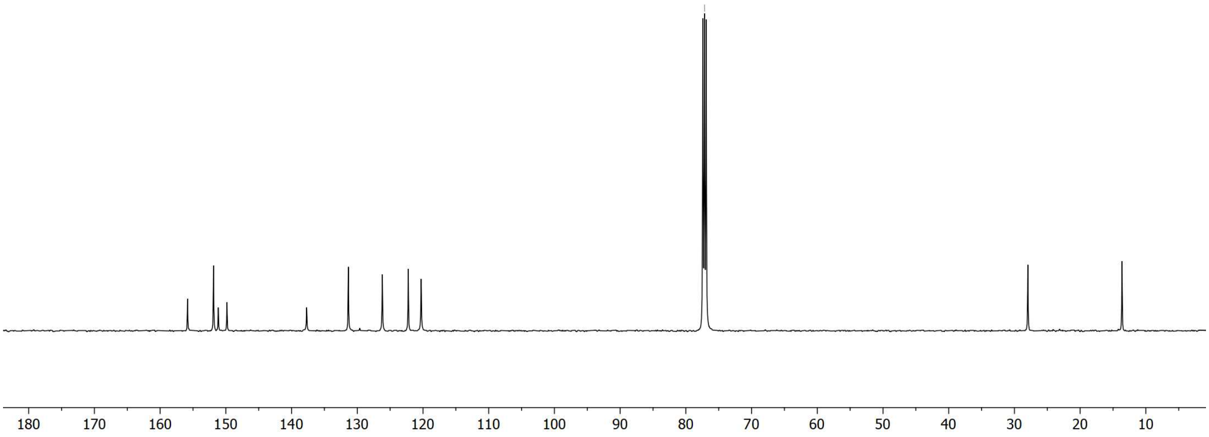

Figure S2. ^{13}C NMR spectrum of **Pt(BPI^{Et})Cl** in $CDCl_3$ (500 MHz).

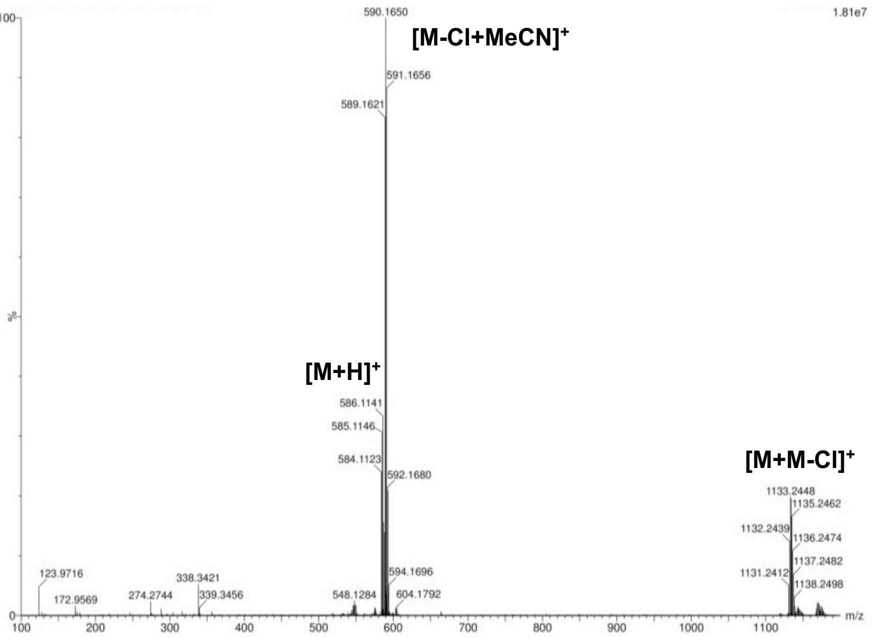

Figure S3. HRMS spectrum of **Pt(BPI^{Et})Cl**.

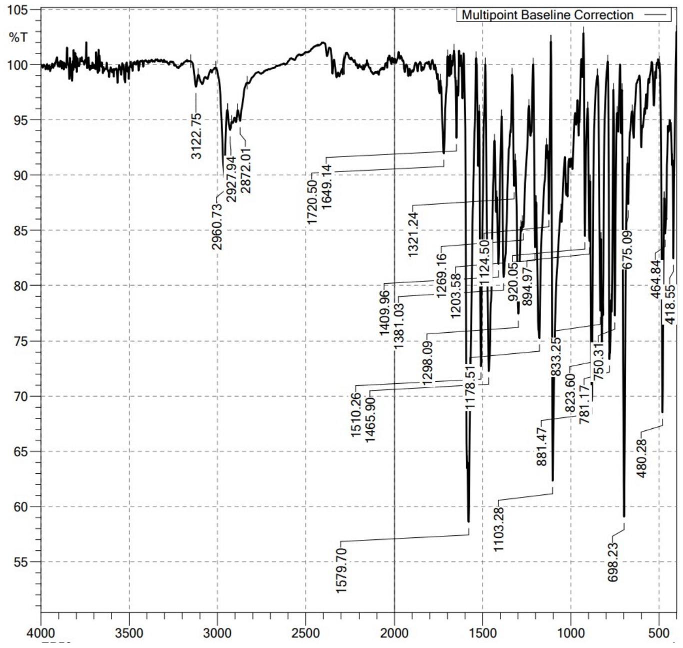

Figure S4. Infrared spectrum of **Pt(BPI^{Et})Cl**.

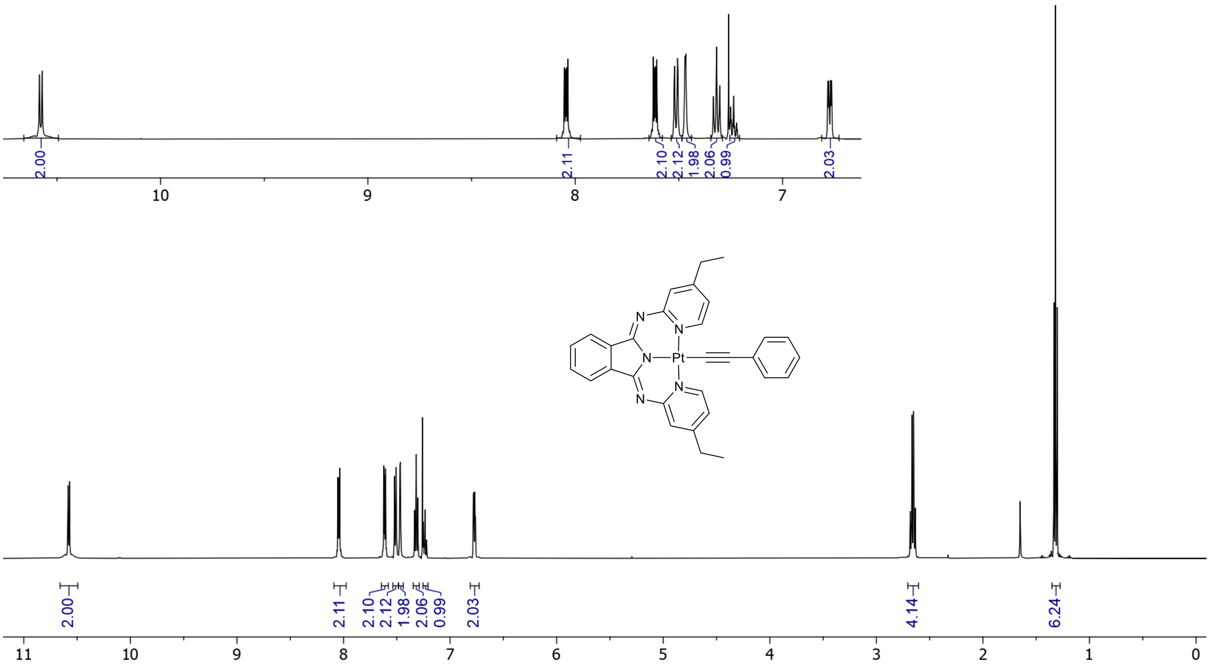

Figure S5. ^{1}H NMR spectrum of **Pt(BPI^{Et})(1)** in $CDCl_3$ (500 MHz).

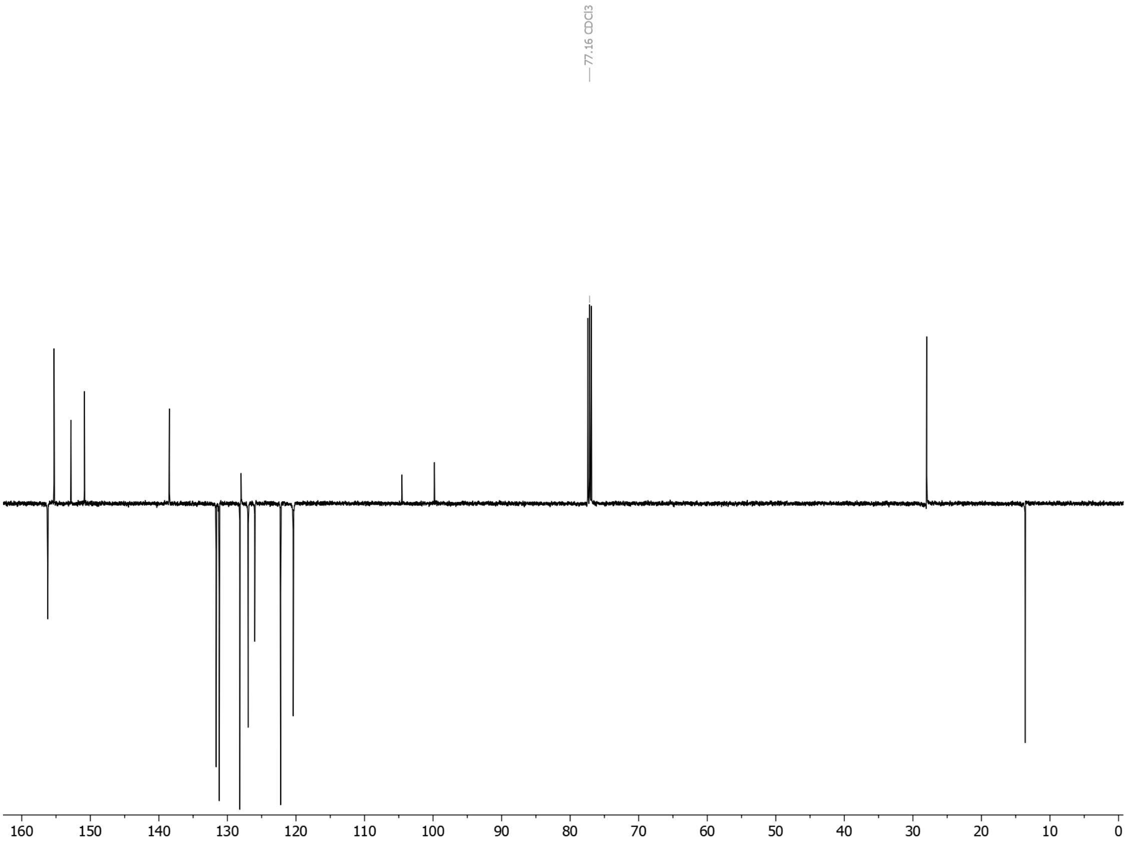

Figure S6. ^{13}C NMR spectrum of **Pt(BPI^{Et})(1)** in $CDCl_3$ (500 MHz).

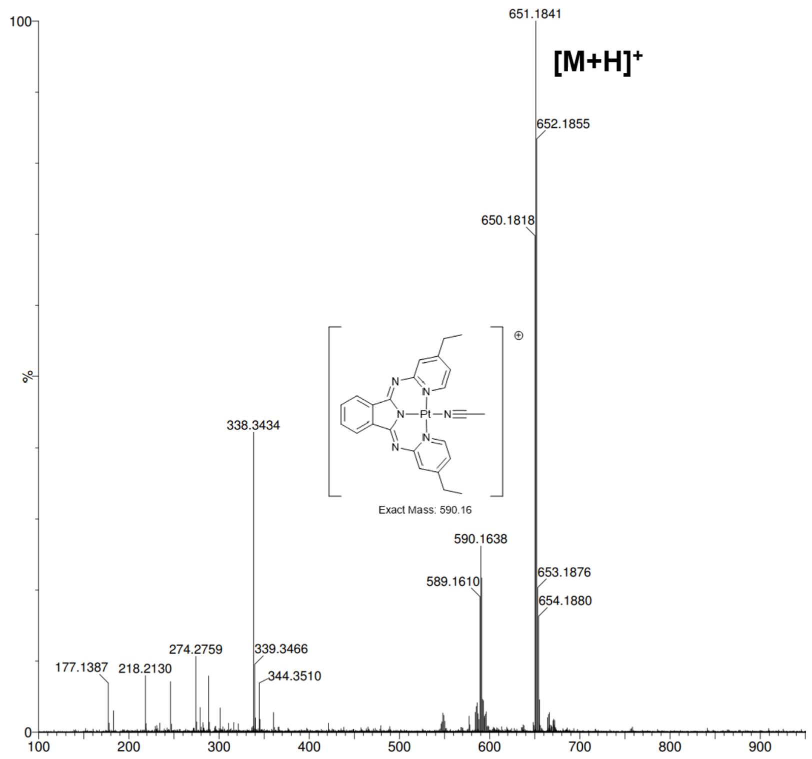

Figure S7. HRMS spectrum of **Pt(BPI^{Et})(1)**.

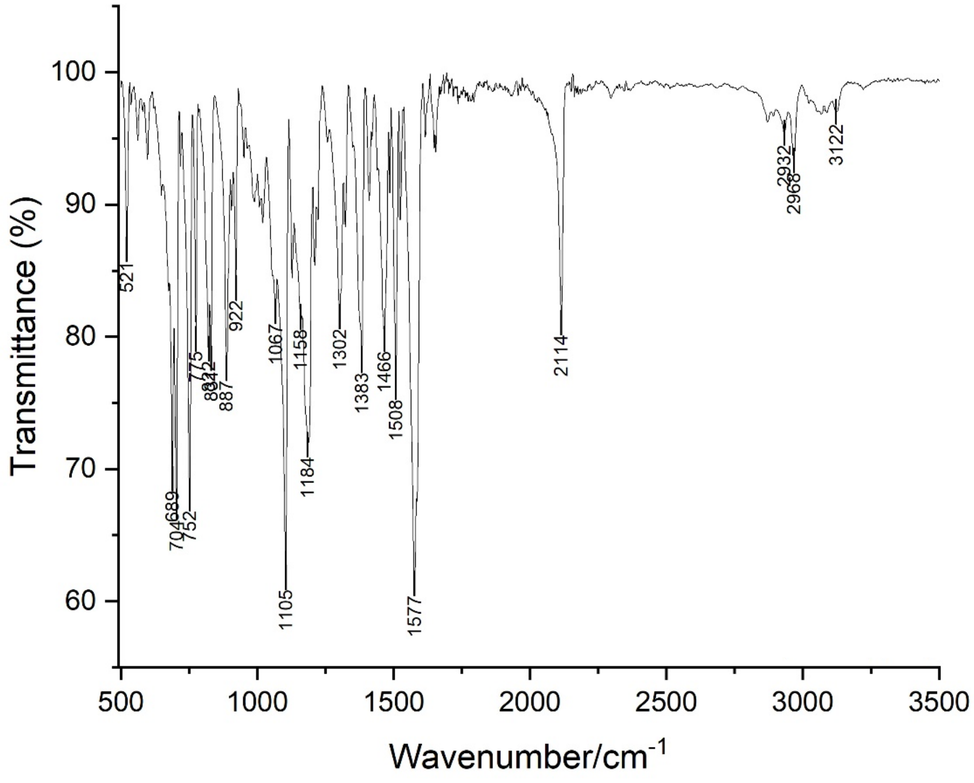

Figure S8. Infrared spectrum of **Pt(BPI^{Et})(1)**.

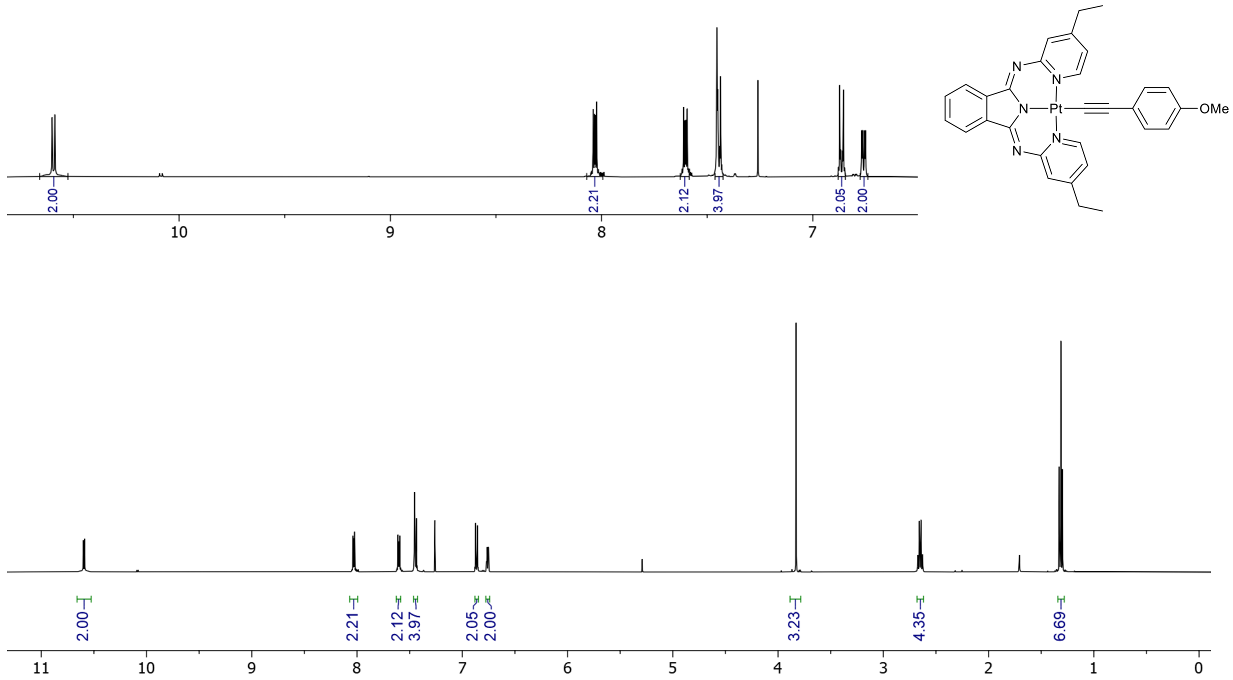

Figure S9. ^{1}H NMR spectrum of **Pt(BPI^{Et})(2)** in $CDCl_3$ (500 MHz). Expansion shows aromatic region.

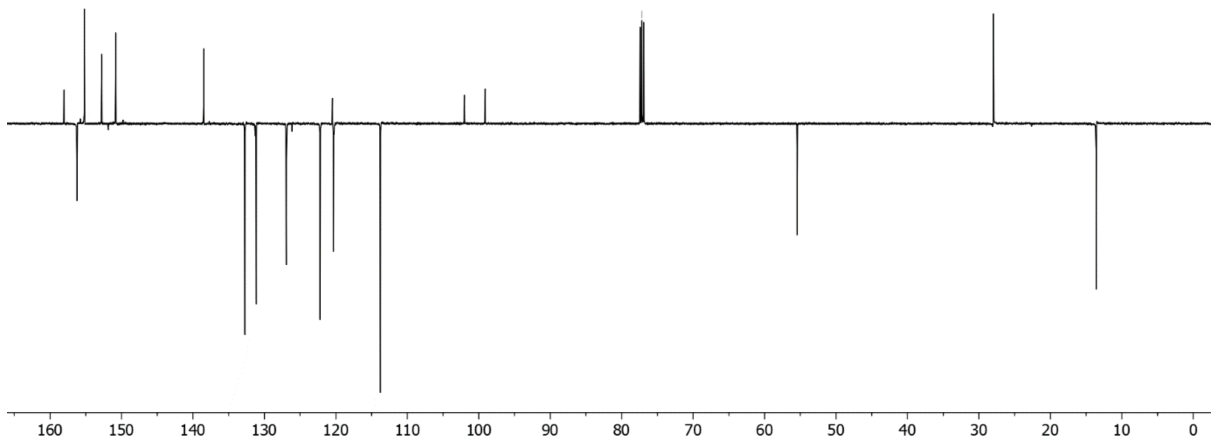

Figure S10. ^{13}C NMR spectrum of **Pt(BPI^{Et})(2)** in $CDCl_3$ (500 MHz).

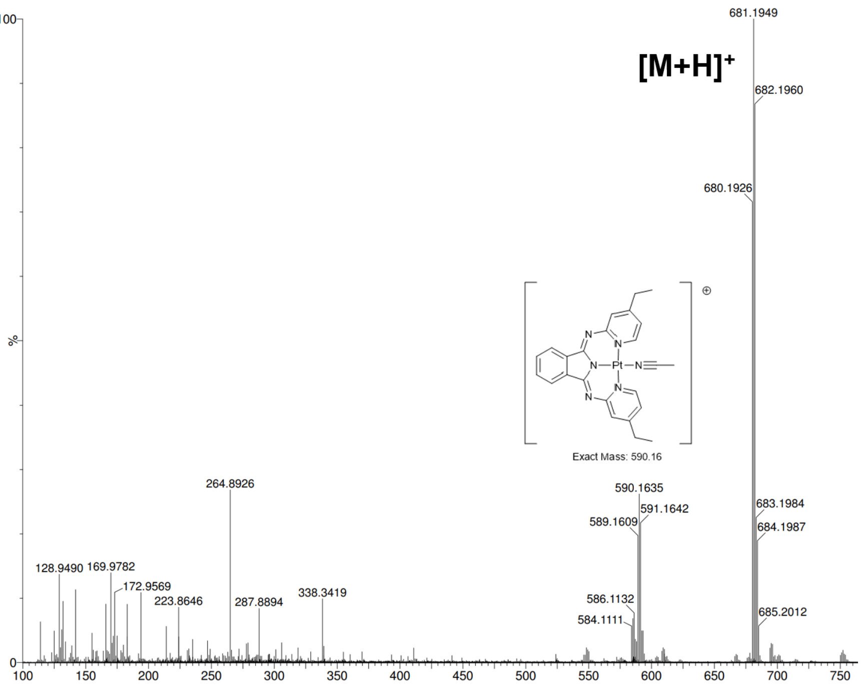

Figure S11. HRMS spectrum of **Pt(BPI^{Et})(2)**.

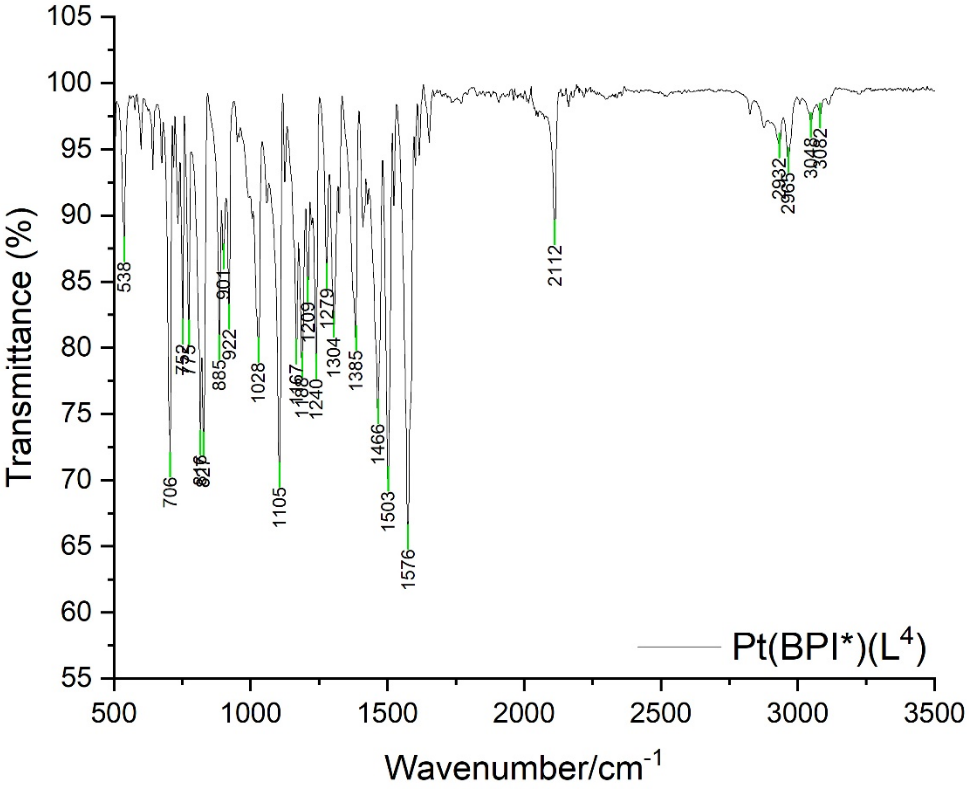

Figure S12. Infrared spectrum of **Pt(BPI^{Et})(2)**.

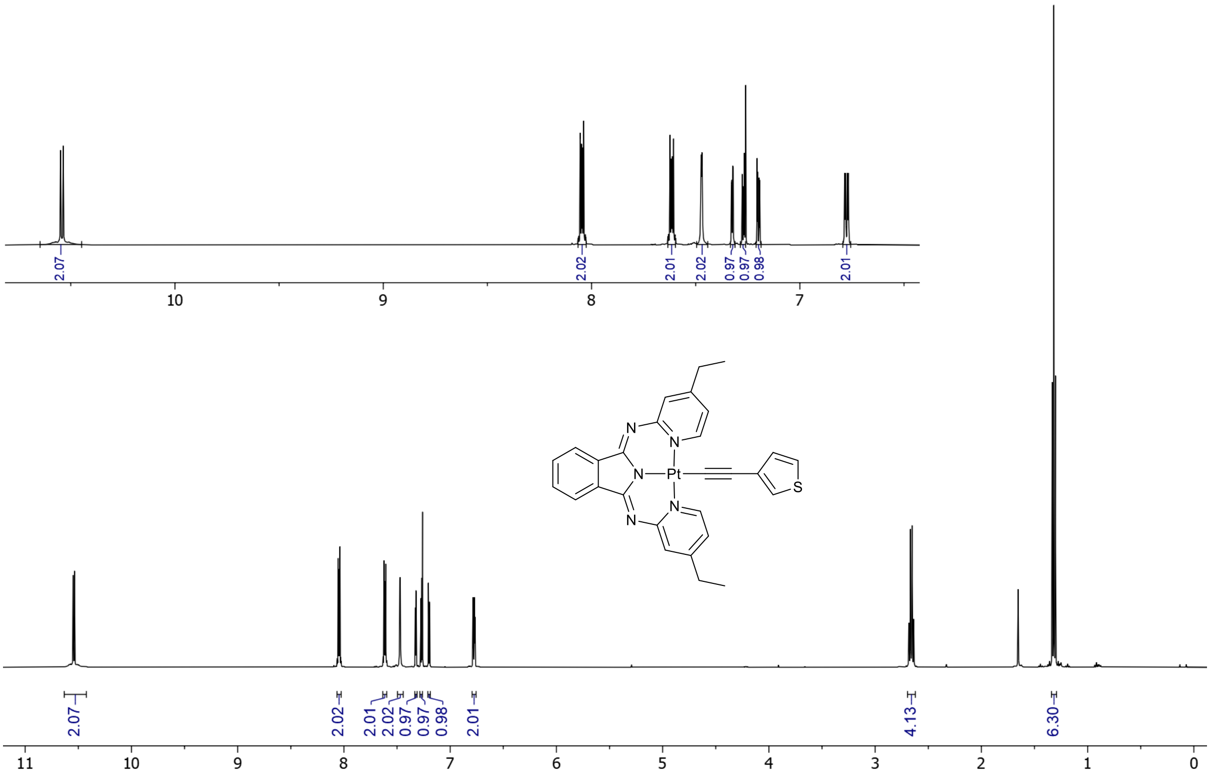

Figure S13. ^{1}H NMR spectrum of **Pt(BPI^{Et})(3)** in $CDCl_3$ (500 MHz). Expansion shows aromatic region.

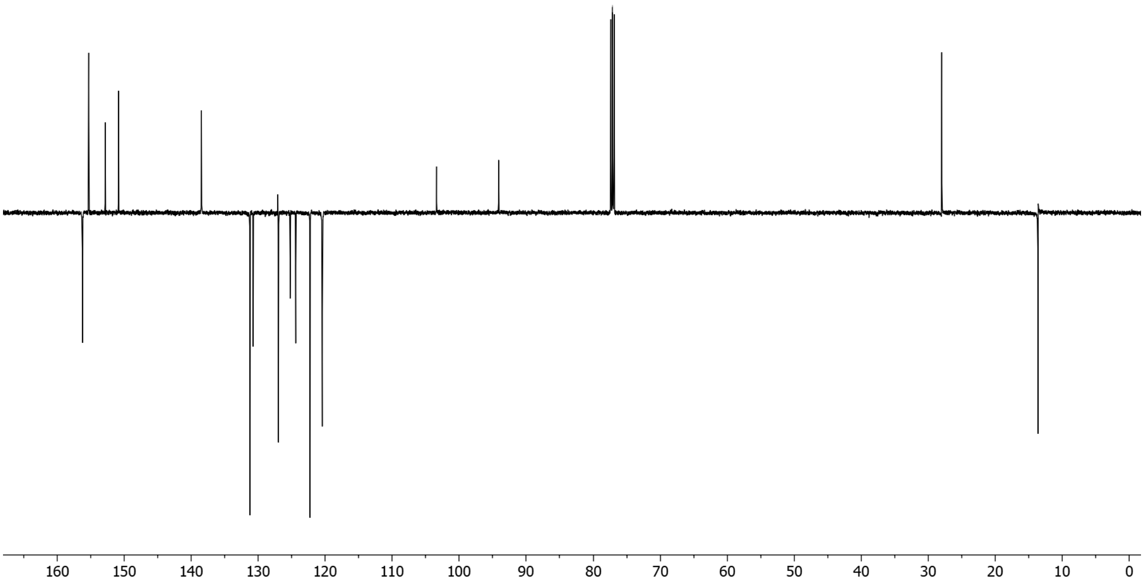

Figure S14. ^{13}C NMR spectrum of **Pt(BPI^{Et})(3)** in $CDCl_3$ (500 MHz).

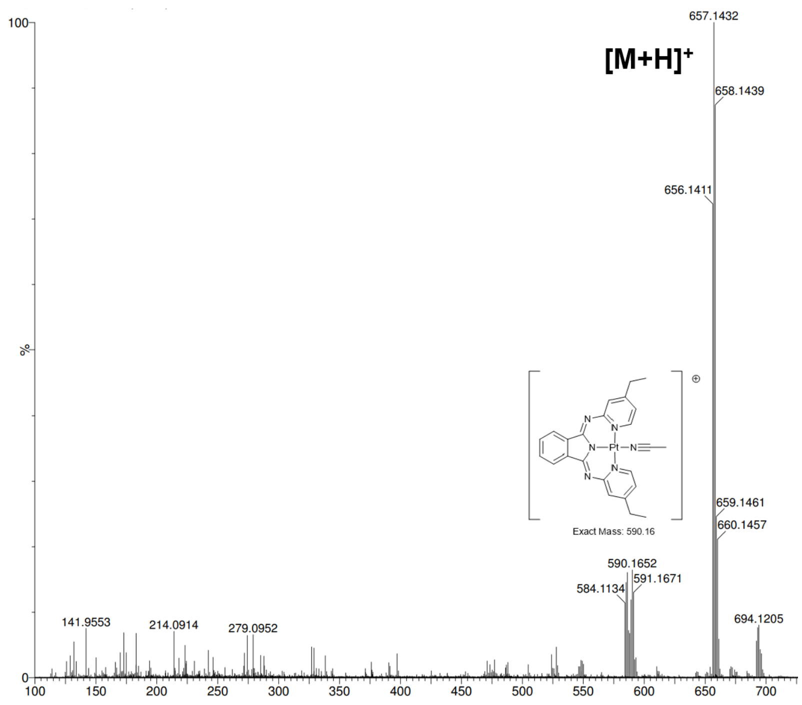

Figure S15. HRMS spectrum of **Pt(BPI^{Et})(3)**.

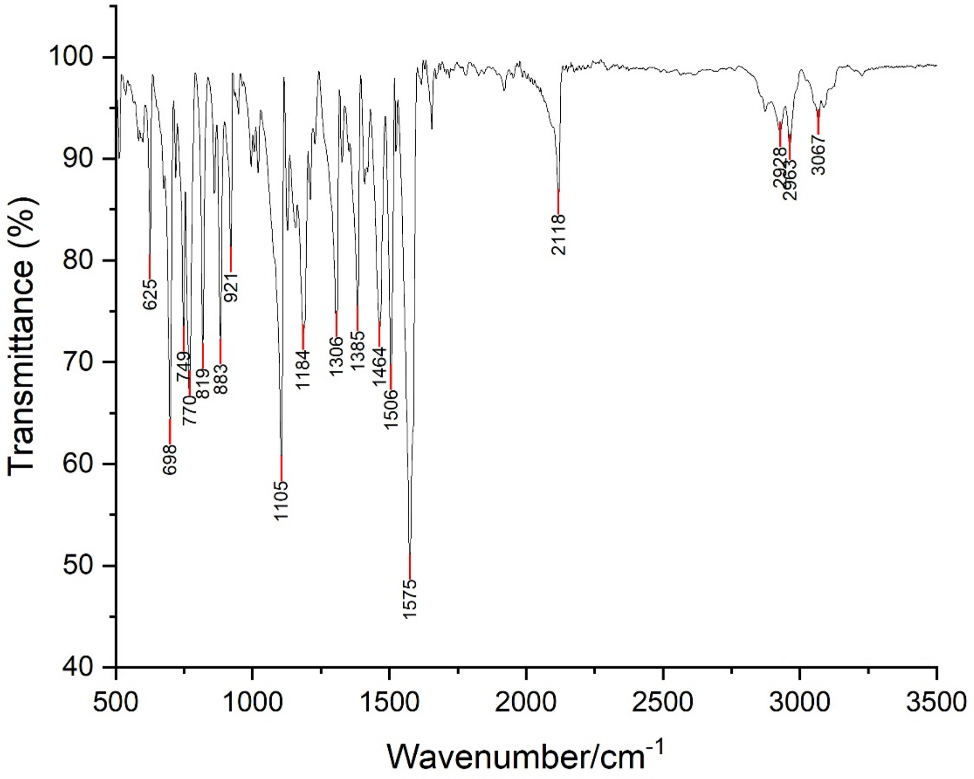

Figure S16. Infrared spectrum of **Pt(BPI^{Et})(3)**.

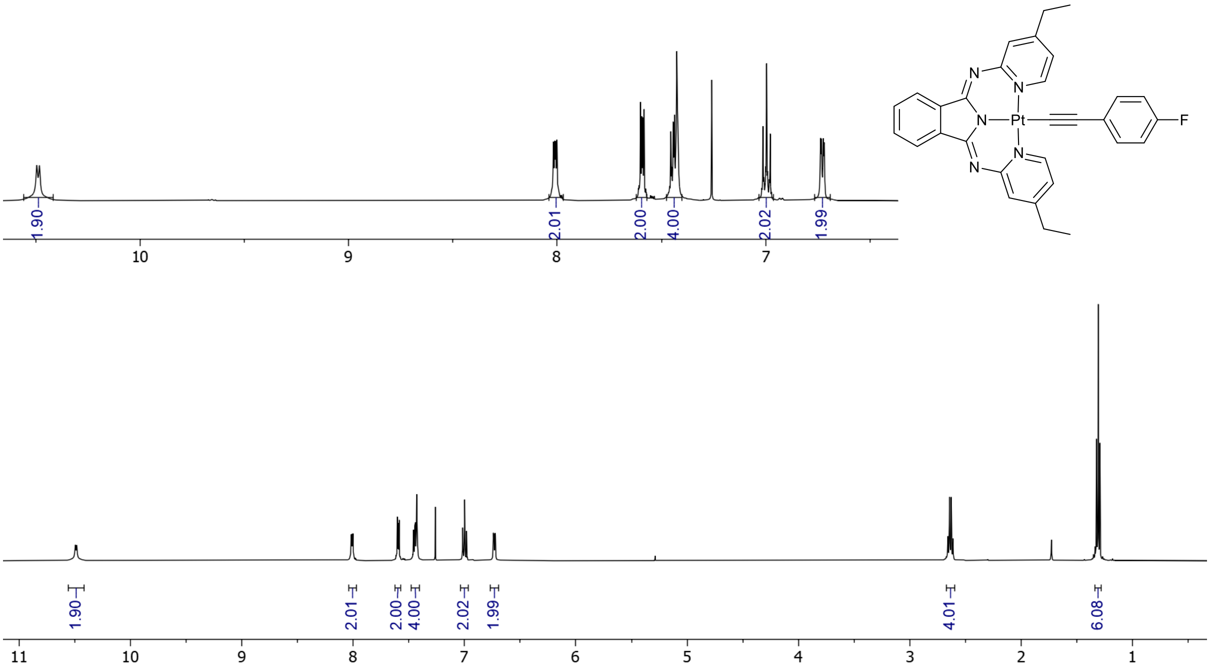

Figure S17. ^{1}H NMR spectrum of **Pt(BPI^{Et})(4)** in $CDCl_3$ (500 MHz). Expansion shows aromatic region.

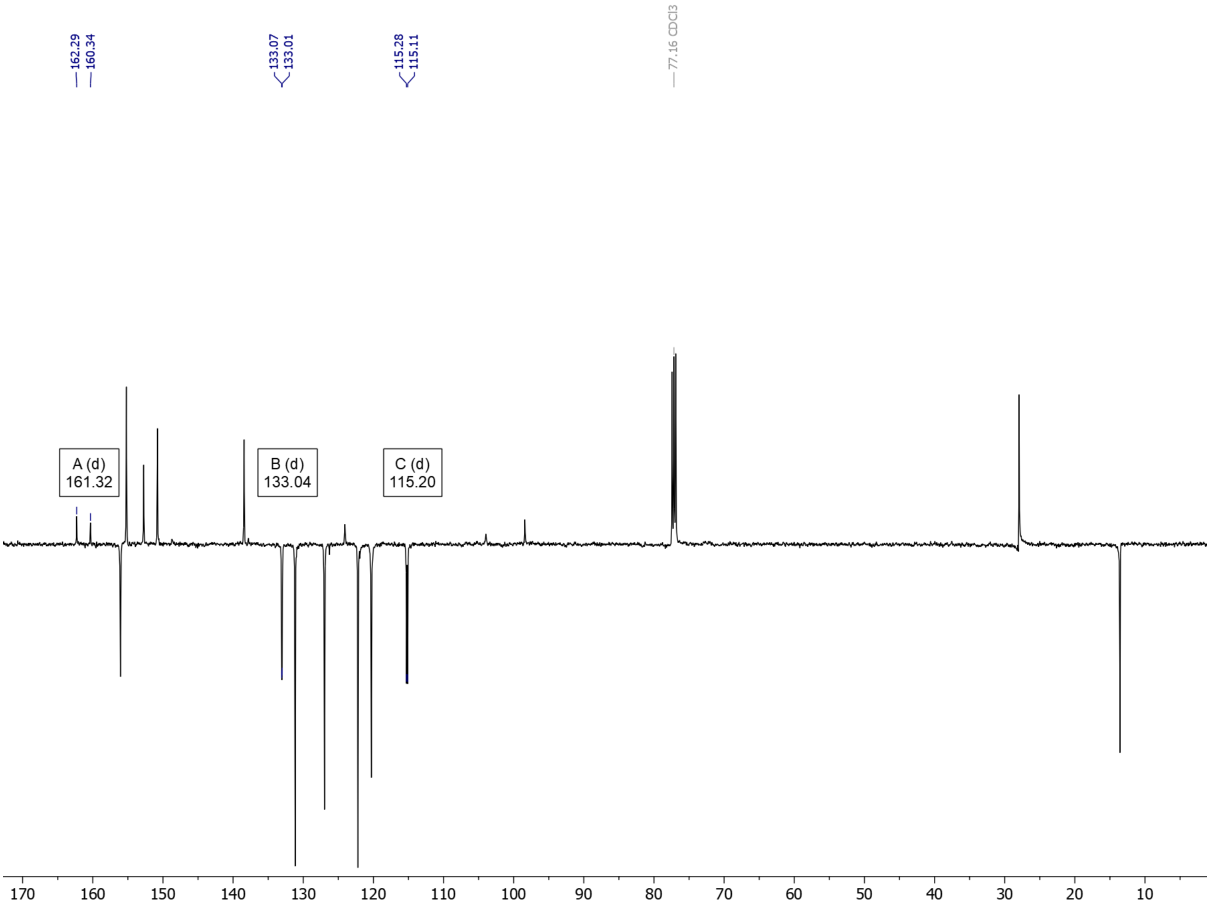

Figure S18. ^{13}C NMR spectrum of **Pt(BPI^{Et})(4)** in $CDCl_3$ (125 MHz).

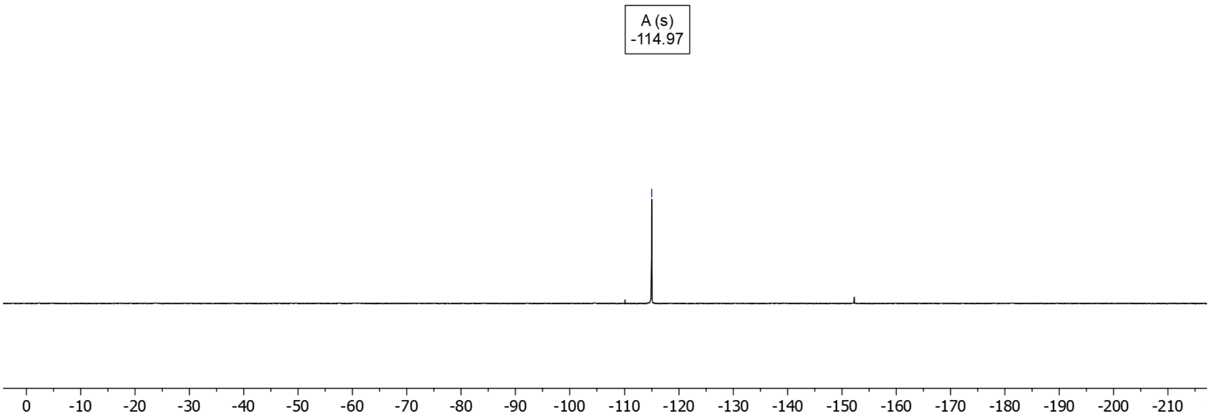

Figure S19. ^{19}F NMR spectrum of **Pt(BPI^{Et})(4)** in $CDCl_3$ (377 MHz).

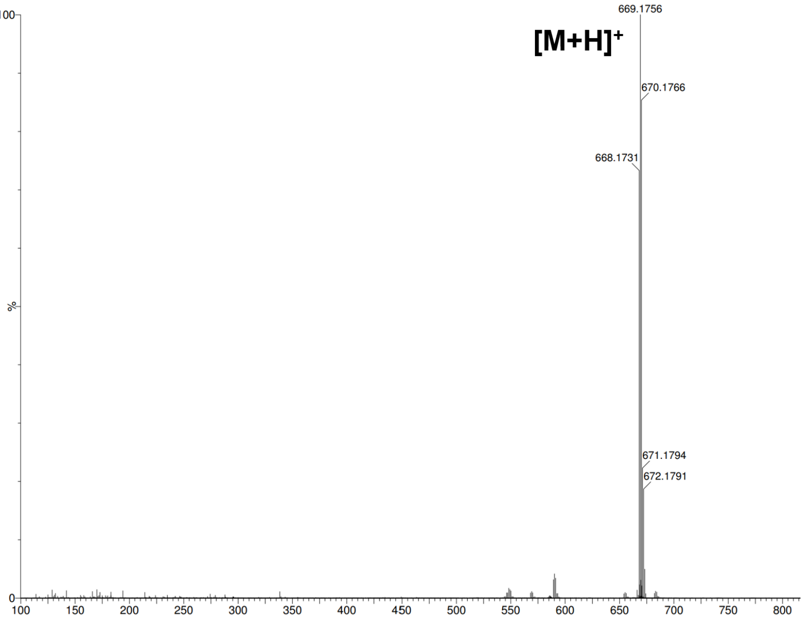

Figure S20. HRMS spectrum of **Pt(BPI^{Et})(4)**.

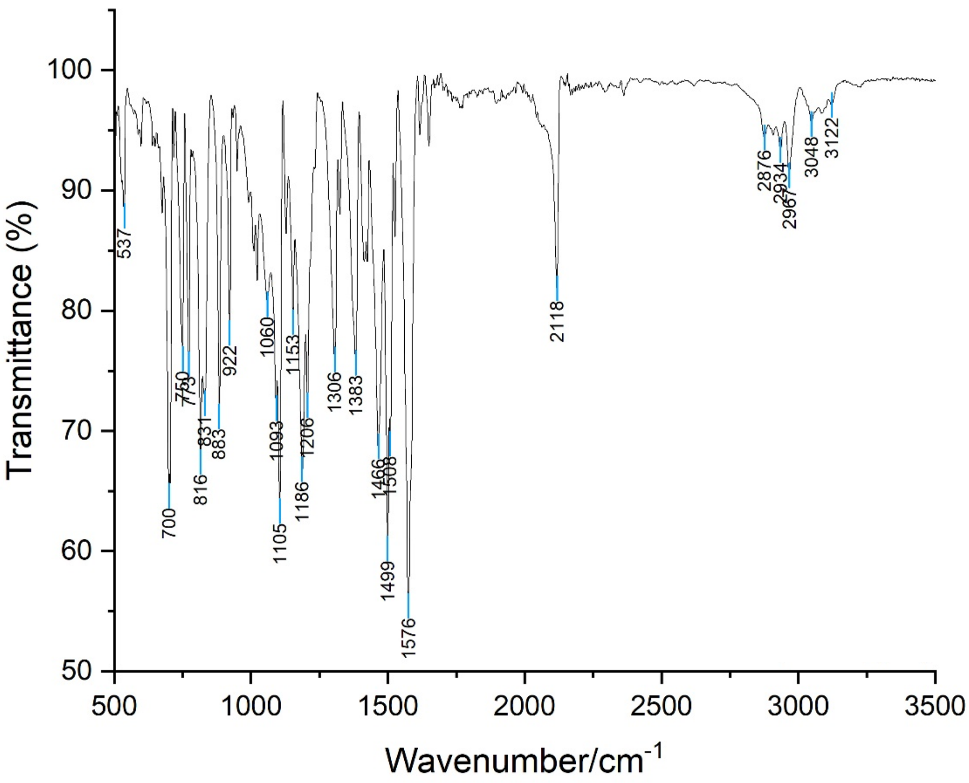

Figure S21. Infrared spectrum of **Pt(BPI^{Et})(4)**.

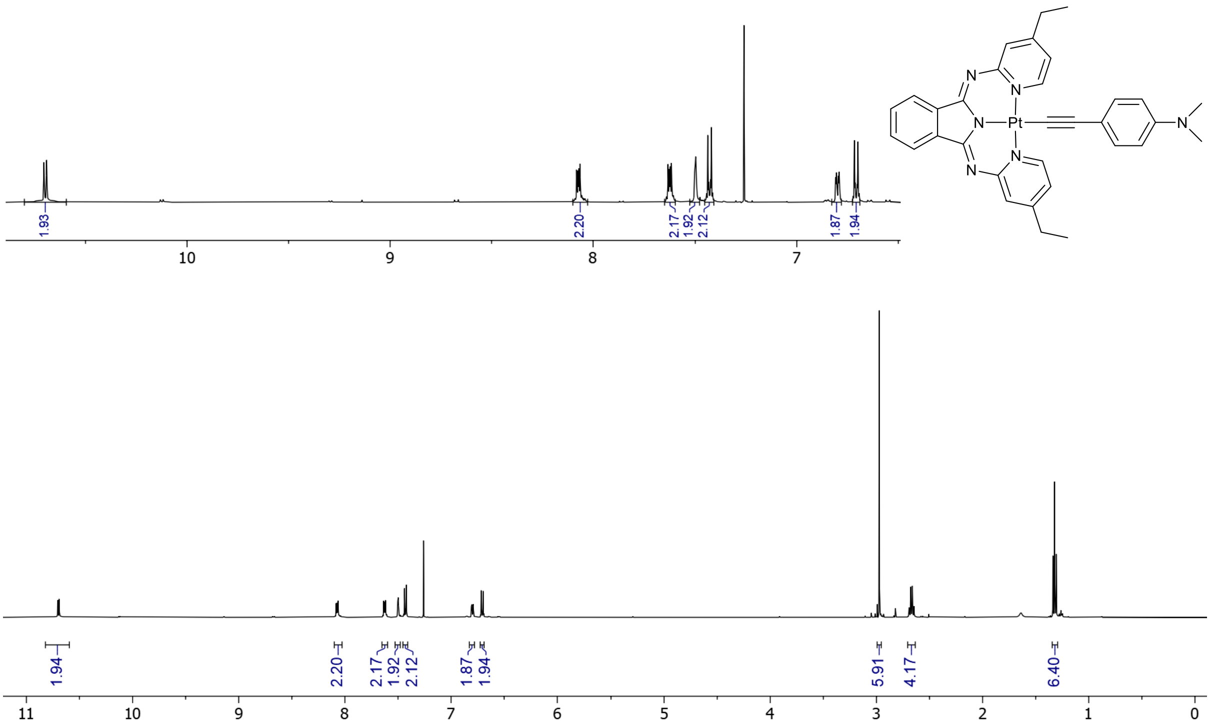

Figure S22. ^{1}H NMR spectrum of **Pt(BPI^{Et})(5)** in $CDCl_3$ (500 MHz). Expansion shows aromatic region.

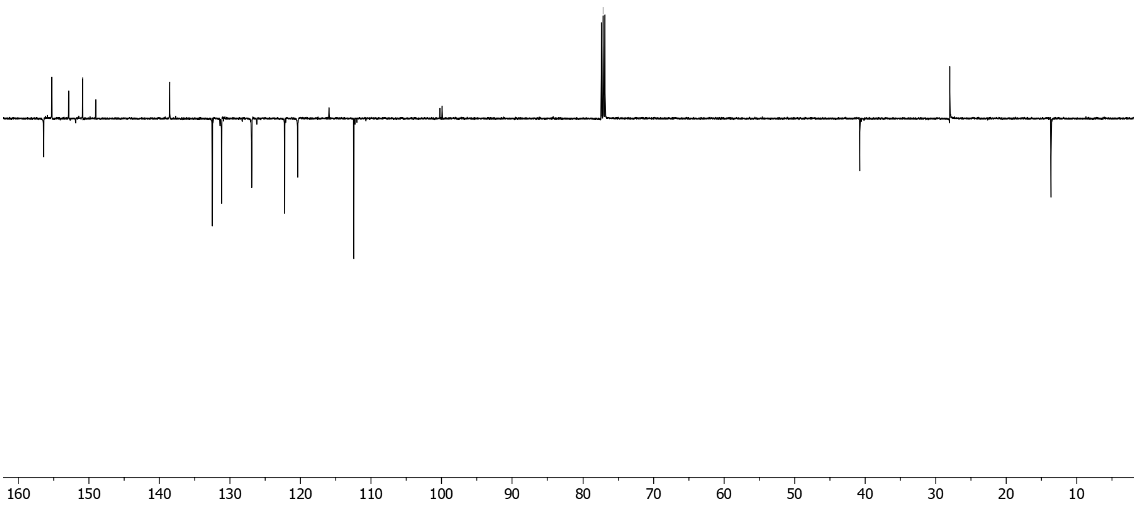

Figure S23. ^{13}C NMR spectrum of **Pt(BPI^{Et})(5)** in $CDCl_3$ (500 MHz).

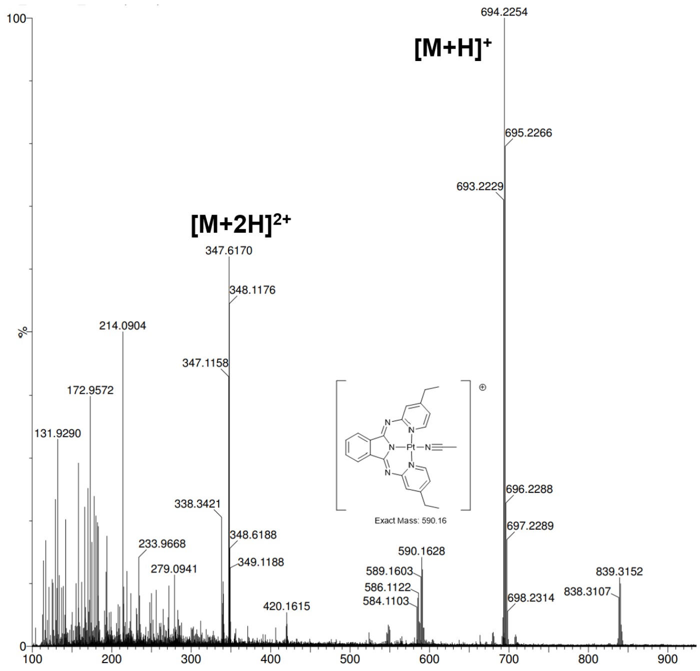

Figure S24. HRMS spectrum of **Pt(BPI^{Et})(5)**.

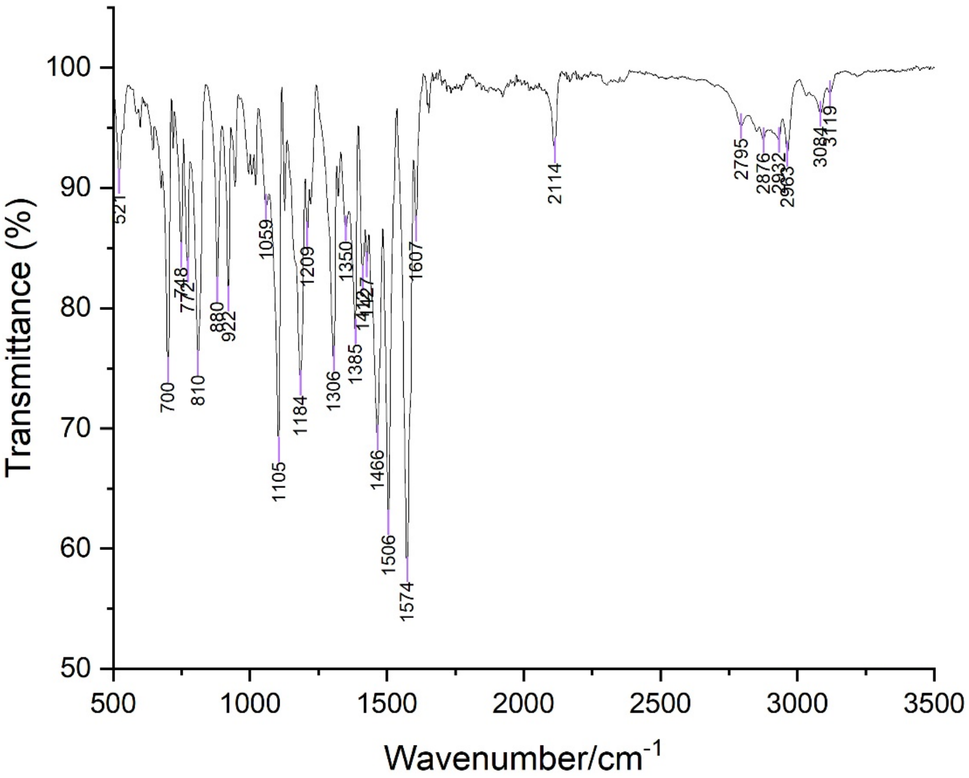

Figure S25. Infrared spectrum of **Pt(BPI^{Et})(5)**.

Table S1. Data collection parameters for the X-ray crystal structures.

	$Pt(BPI^{Et})(2)$	$Pt(BPI^{Et})(3)$
Formula	$C_{31}H_{27}N_5OPt$	$C_{28}H_{23}N_5SPt$
$D_{calc.}$/ g cm^{-3}	1.782	1.847
μ/mm^{-1}	5.563	6.056
Formula Weight	680.671	656.672
Colour	red	red
Shape	rod-shaped	plate-shaped
Size/mm^3	0.107×0.046×0.024	0.111×0.093×0.021
T/K	100(2)	100(2)
Crystal System	monoclinic	monoclinic
Space Group	$C2/c$	Pc
a/Å	21.4630(6)	12.6607(1)
b/Å	12.8778(3)	12.9128(1)
c/Å	18.8912(5)	7.3668(1)
α/°	90	90
β/°	103.580(3)	101.330(1)
γ/°	90	90
V/Å^3	5075.5(2)	1180.89(2)
Z	8	2
Z'	1	1
Wavelength/Å	0.71075	0.71075
Radiation type	Mo K_α	Mo K_α
Θ_{min}/°	1.86	2.28
Θ_{max}/°	30.51	37.79
Measured Refl's.	118579	94927
Indep't Refl's	7748	12121
Refl's I≥2 σ(I)	6177	10660
R_{int}	0.0874	0.0519
Parameters	411	390
Restraints	1065	265
Largest Peak	1.2147	1.4512
Deepest Hole	-0.8764	-1.2820
GooF	1.0360	0.9875
wR_2 (all data)	0.0803	0.0784
wR_2	0.0747	0.0753
R_1 (all data)	0.0459	0.0423
R_1	0.0330	0.0333

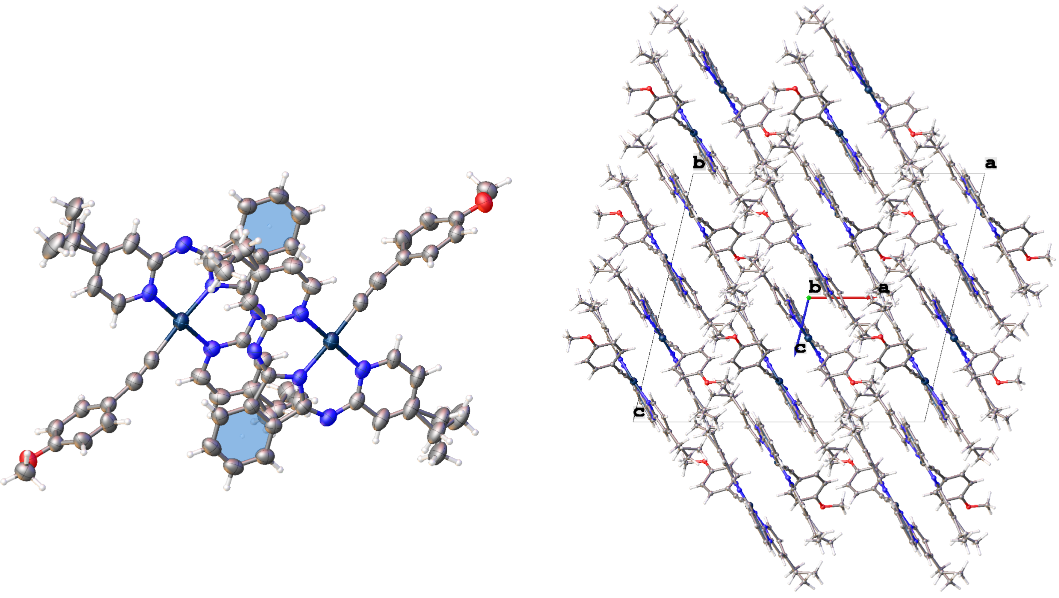

Figure S26. Packing diagrams obtained from the X-ray structure of **Pt(BPI^{Et})(2)**. View along the b axis shown, right.

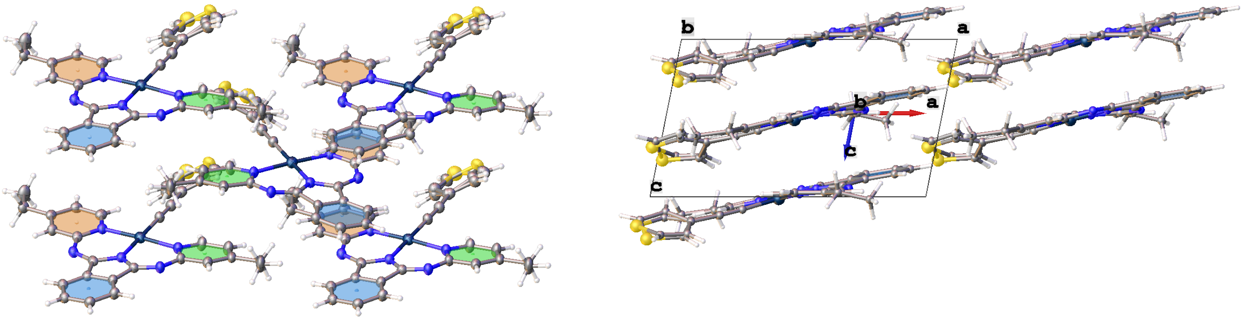

Figure S27. Packing diagrams obtained from the X-ray structure of **Pt(BPI^{Et})(3)**. View along the b axis shown, right.

Table S2. Comparison of experimental (X-ray) and calculated (DFT; values in italics) parameters that describe the coordination geometries.

Bond lengths (Å)

**Pt(BPI^{Et})(2)**			**Pt(BPI^{Et})(3)**		
Pt1	N1	2.057(3)<br>*2.074*	Pt1	N1	2.043(3)<br>*2.071*
Pt1	N3	1.992(3)<br>*2.026*	Pt1	N3	2.004(3)<br>*2.025*
Pt1	N5	2.034(3)<br>*2.074*	Pt1	N5	2.075(3)<br>*2.072*
Pt1	C31	2.019(4)<br>*1.977*	Pt1	C31	1.969(4)<br>*1.976*

Bond angles (º)

**Pt(BPI^{Et})(2)**				**Pt(BPI^{Et})(3)**			
N3	Pt1	N1	89.36(13)<br>*89.2*	N3	Pt1	N1	89.44(12)<br>*89.2*
N5	Pt1	N1	175.51(11)<br>*178.1*	N5	Pt1	N1	177.40(10)<br>*175.6*
C31	Pt1	N1	90.34(14)<br>*90.8*	C31	Pt1	N1	91.69(13)<br>*90.8*
C31	Pt1	N3	174.21(14)<br>*178.7*	C31	Pt1	N3	178.12(12)<br>*175.0*
C31	Pt1	N5	90.98(15)<br>*90.8*	C31	Pt1	N5	90.31(13)<br>*91.1*

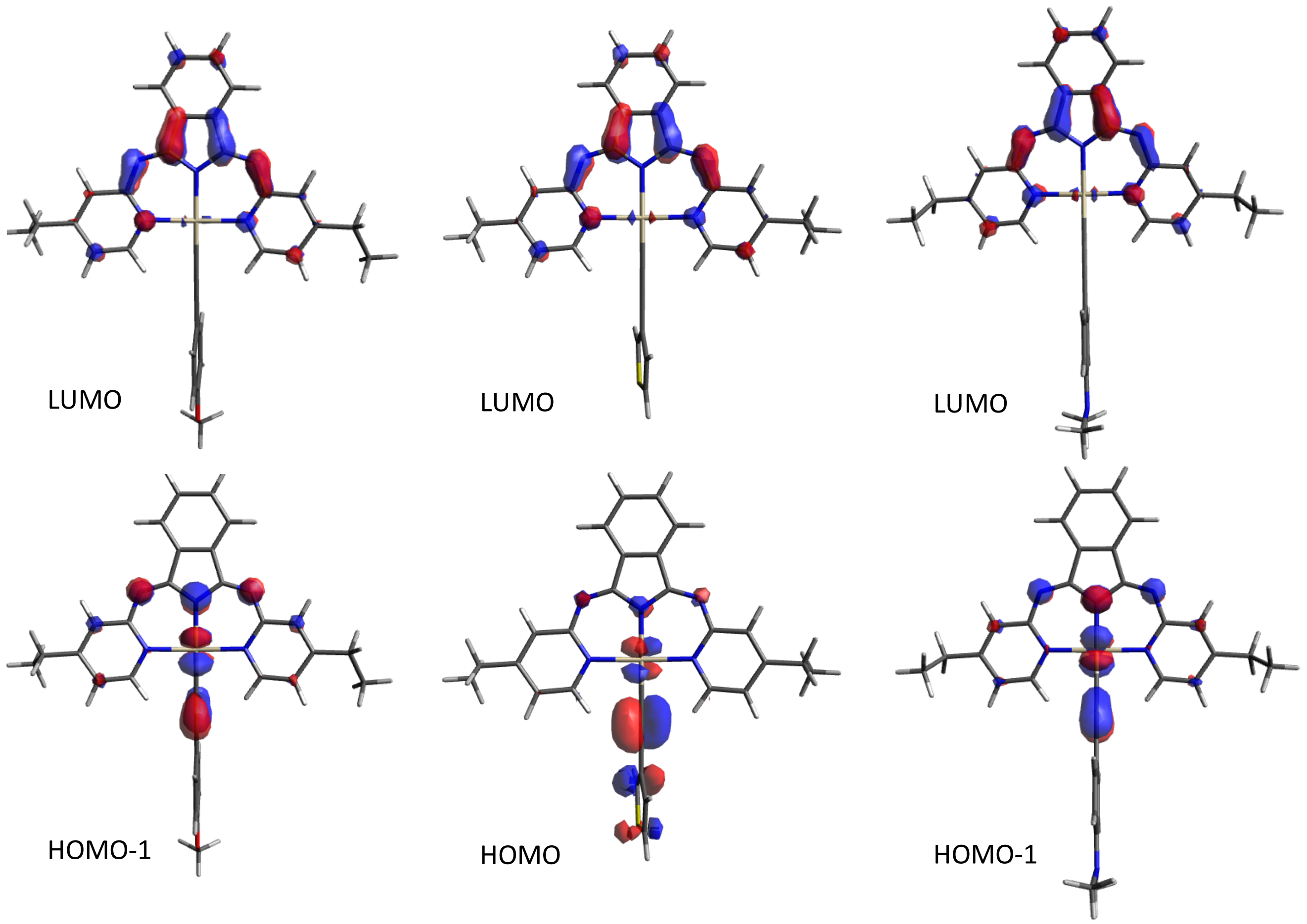

Figure S28. Comparison of the pictorial representation of the frontier orbitals for **Pt(BPI^{Et})(2)** (left) and **Pt(BPI^{Et})(3)** (center) and **Pt(BPI^{Et})(5)** (right).

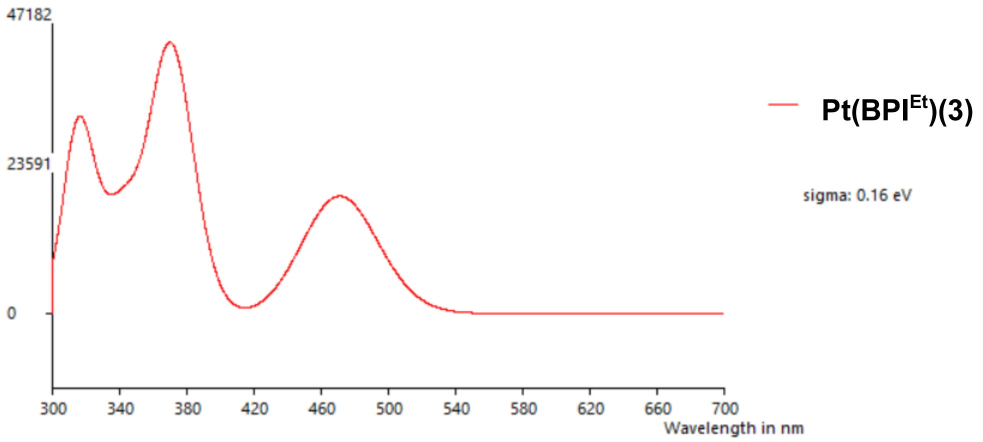

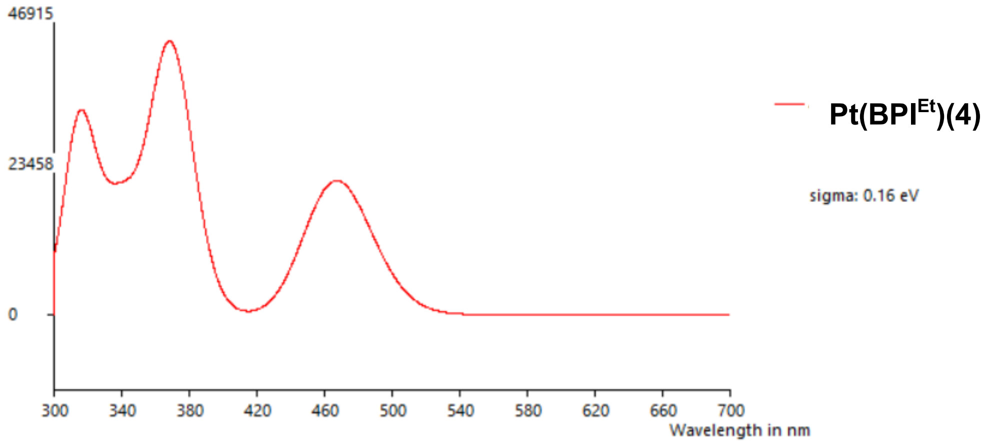

Figure S29. Examples of calculated absorption spectra.

Table S3. Photoluminescence data for the Pt(II) complexes in various solvents.[a]

	λ_{em} / nm[b]				τ / μs[c]				degas τ / μs[d]			
	n-hexane	toluene	chloroform	acetonitrile	n-hexane	toluene	chloroform	acetonitrile	n-hexane	toluene	chloroform	acetonitrile
Pt(1)	619	625	629	632	0.23	0.28	0.31	0.18	1.3	1.1, 1.5	1.0	0.4, 0.69
Pt(2)	632	644	644	661	0.14	0.13	0.007	0.01, 0.17	1.0, 4.8	0.24, 1.1	0.007, 0.84	0.66
Pt(3)	619	630	638	644	0.21	0.24	0.004, 0.1	0.1	0.7, 0.9	0.79, 1.4	0.13	0.18, 0.76
Pt(4)	615	624	636	636	0.23	0.28	0.23	0.18	1.1, 1.5	0.93	0.005, 0.42	0.64
Pt(5)	670	644	621	641	0.01, 0.19	0.26	0.004, 0.05	0.18	0.95	0.55, 1.1	0.006, 0.86	0.59, 1.1

[a] All measurements obtained at 293 K unless otherwise stated using *A* = 0.1

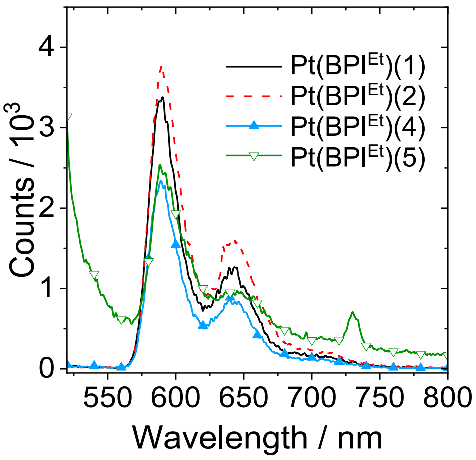

Figure S30. Low temperature photoluminescence emission spectra of selected complexes in frozen 2-MeTHF glass (77 K). Excited with a picosecond pulsed laser (510 nm, c = 1.0 × 10^{-4} M, 77 K).

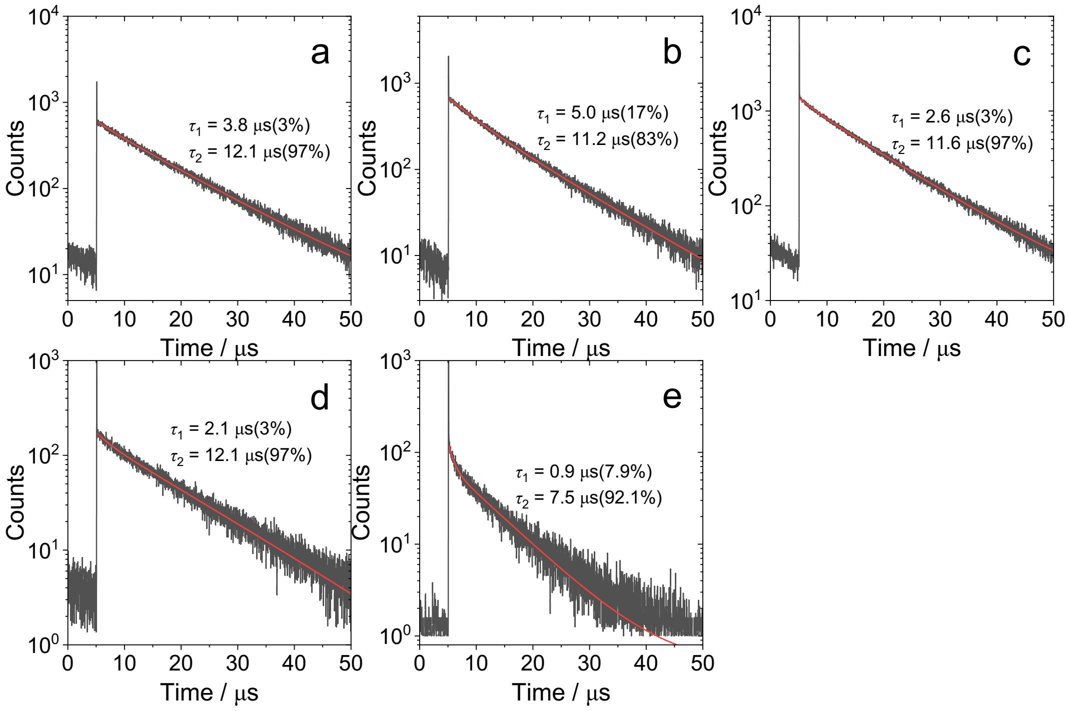

Figure S31. Low temperature lifetime spectra of the complexes (a) **Pt(BPI^{Et})(1)** at 590 nm, (b) **Pt(BPI^{Et})(2)** at 590 nm, (c) **Pt(BPI^{Et})(3)** at 588 nm, (d) **Pt(BPI^{Et})(4)** at 589 nm, (e) **Pt(BPI^{Et})(5)** at 588 nm in frozen 2-MeTHF glass (77 K). Excited with a picosecond pulsed laser (510 nm).

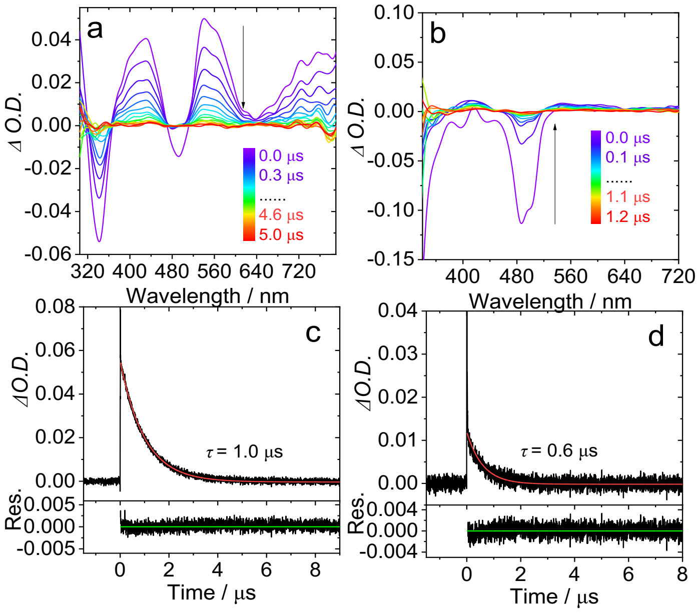

Figure S32. Nanosecond transient absorption spectra of (a) **Pt(BPI^{Et})(4)**, $c = 3.1 \times 10^{-5}$ M excited at 480 nm, (b) **Pt(BPI^{Et})(5)**, $c = 5.3 \times 10^{-5}$ M excited at 470 nm in deaerated *n*-hexane. The decay traces of (c) **Pt(BPI^{Et})(4)**, $c = 3.1 \times 10^{-5}$ M, excited at 480 nm, (d) **Pt(BPI^{Et})(5)**, $c = 5.3 \times 10^{-5}$ M, λ_{em} =545 nm, excited at 470 nm in deaerated *n*-hexane.

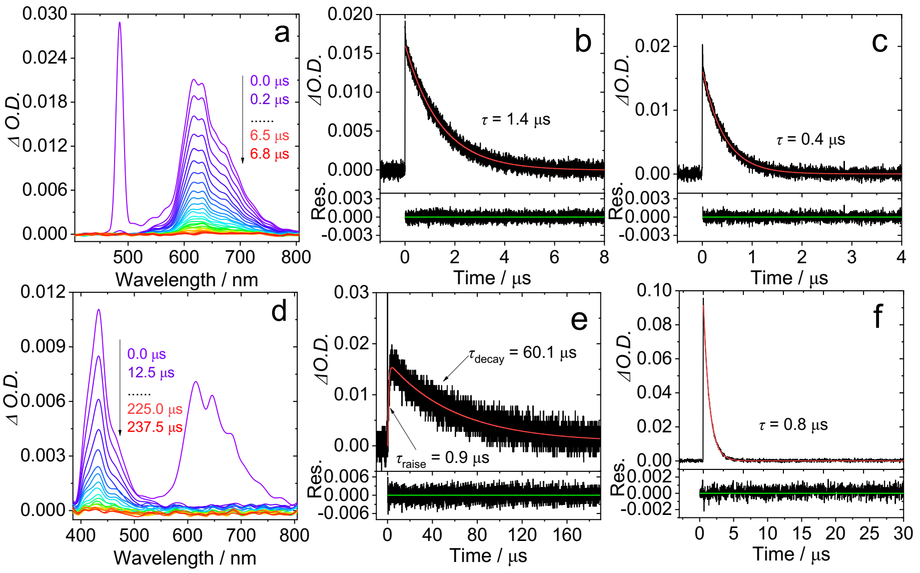

Figure S33. (a) Time-resolved luminescence of **Pt(BPI^{Et})(1)** ($c = 1.3 \times 10^{-5}$ M); (b) the decay traces of phosphorescence in N_2; (c) the decay traces of phosphorescence in air; (d) delayed fluorescence with **Pt(BPI^{Et})(1)** ($c = 1.3 \times 10^{-5}$ M) as the triplet photosensitizer and DPA ($c = 2.0 \times 10^{-4}$ M) as the triplet acceptor; (e) the decay traces of the emission at 430 nm (^{1}DPA* → S_0); (f) the decay traces of the emission at 600 nm ($T_1 \rightarrow S_0$) ; the spike in the delayed fluorescence traces is the scattered laser. Excited with nanosecond pulsed laser at 480 nm. In deaerated toluene, 20°C.

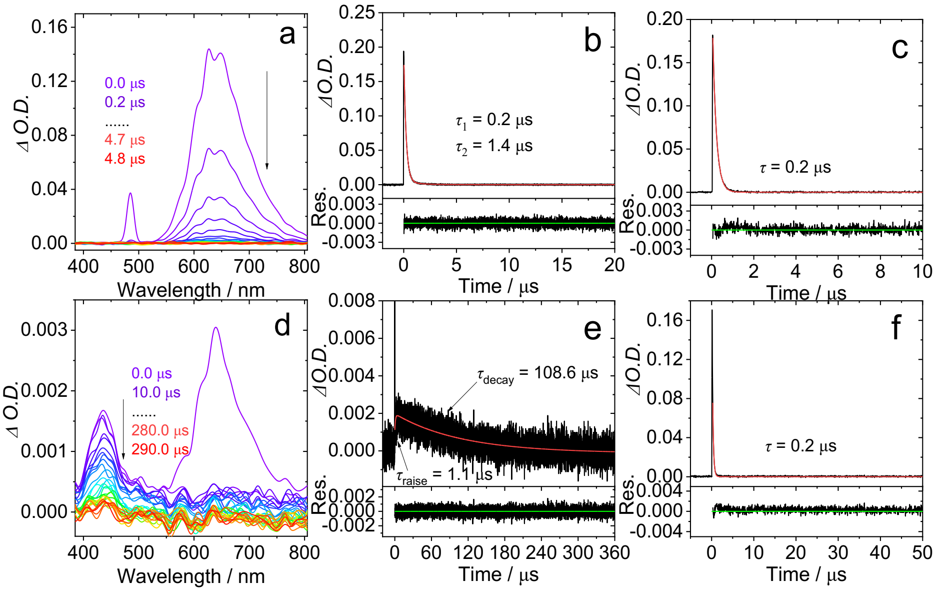

Figure S34. (a) Time-resolved luminescence of **Pt(BPI^{Et})(2)** (c = 2 × 10^{-5} M); (b) the decay traces of phosphorescence in N_2; (c) the decay traces of phosphorescence in air; (d) delayed fluorescence with **Pt(BPI^{Et})(2)** (c = 2 × 10^{-5} M) as the triplet photosensitizer and DPA (c = 2.0 × 10^{-4} M) as the triplet acceptor; (e) the decay traces of the emission at 430 nm (^{1}DPA* → S_0); (f) the decay traces of the emission at 635 nm (T_1 → S_0); the spike in the delayed fluorescence traces is the scattered laser. Excited with nanosecond pulsed laser at 480 nm. In deaerated toluene, 20 °C.

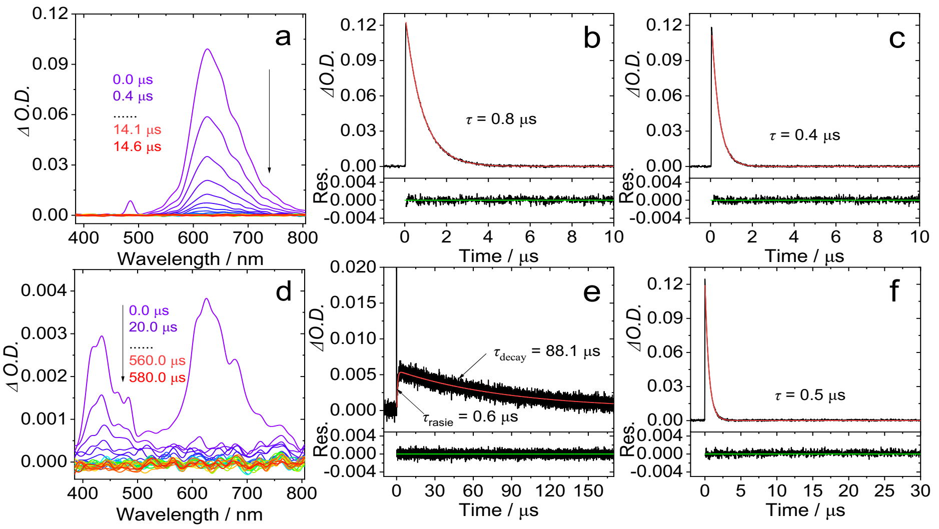

Figure S35. (a) Time-resolved luminescence of **Pt(BPI^{Et})(3)** (c = 1 × 10^{-5} M); (b) the decay traces of phosphorescence in N_2; (c) the decay traces of phosphorescence in air; (d) delayed fluorescence with **Pt(BPI^{Et})(3)** (c = 1 × 10^{-5} M) as the triplet photosensitizer and DPA (c = 2.0 × 10^{-4} M) as the triplet acceptor; (e) the decay traces of the emission at 430 nm (^{1}DPA* → S_0); (f) the decay traces of the emission at 625 nm (T_1 → S_0); the spike in the delayed fluorescence traces is the scattered laser. Excited with nanosecond pulsed laser at 480 nm. In deaerated toluene, 20 °C.

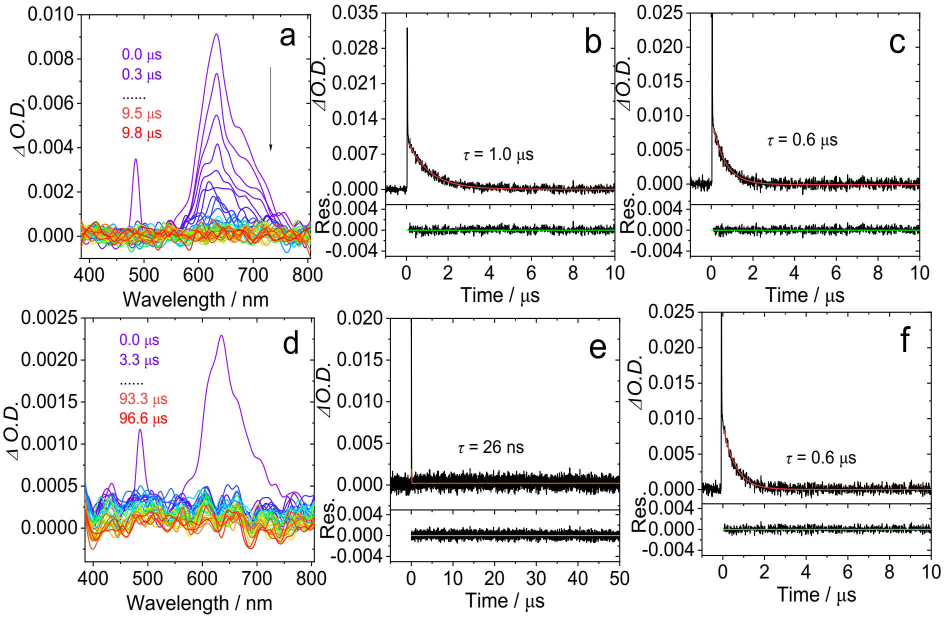

Figure S36. (a) Time-resolved luminescence of **Pt(BPI^{Et})(5)** (c = 2 × 10^{-5} M); (b) the decay traces of phosphorescence in N_2; (c) the decay traces of phosphorescence in air; (d) delayed fluorescence with **Pt(BPI^{Et})(5)** (c = 2 × 10^{-5} M) as the triplet photosensitizer and DPA (c = 2.0 × 10^{-4} M) as the triplet acceptor; (e) the decay traces of the emission at 430 nm (^{1}DPA* → S_0); (f) the decay traces of the emission at 625 nm (T_1 → S_0); the spike in the delayed fluorescence traces is the scattered laser. Excited with nanosecond pulsed laser at 480 nm. In deaerated toluene, 20 °C.

Table S4. Optimized cartesian coordinates

Pt(BPI^{Et})(1)

C	-5.235478	0.158734	1.475574
C	-4.616399	0.148653	0.209601
C	-5.437870	0.188519	-0.934938
C	-6.823677	0.237105	-0.814658
C	-7.422979	0.247083	0.445885
C	-6.621898	0.207525	1.588310
C	-3.192278	0.101173	0.087654
C	-1.967385	0.064109	-0.028590
Pt	0.003909	0.000798	-0.139215
N	-0.037833	-2.066560	-0.225077
C	1.068381	-2.863581	-0.116405
C	0.937591	-4.269981	-0.188210
C	-0.276025	-4.896735	-0.398644
C	-1.387025	-4.049113	-0.538613
C	-1.225151	-2.684652	-0.446710
N	2.350454	-2.438455	0.050932
C	2.759855	-1.214224	0.047827
N	2.027116	-0.071484	-0.088778
C	2.844416	1.016872	0.001567
C	4.232963	0.548974	0.174548
C	4.179441	-0.845546	0.208680

C	5.326046	-1.611812	0.370299
C	6.541068	-0.931809	0.497117
C	6.594872	0.467715	0.462267
C	5.435275	1.231963	0.300493
N	2.527240	2.267446	-0.035866
C	1.273870	2.782843	-0.161858
N	0.106620	2.069898	-0.191590
C	-1.044214	2.775961	-0.324900
C	-1.109415	4.147906	-0.426350
C	0.070397	4.908700	-0.396709
C	1.245025	4.193372	-0.260706
C	0.048655	6.407993	-0.467445
C	-0.309969	7.053145	0.870534
C	-0.410898	-6.391164	-0.444591
C	-0.989614	-6.968241	0.846472
H	-8.511196	0.285517	0.537674
H	-1.953942	2.178543	-0.347852
H	-2.088951	4.618271	-0.534615
H	2.216113	4.689137	-0.228744
H	5.465720	2.323109	0.273526
H	7.560858	0.968139	0.564456
H	7.465928	-1.499202	0.626130
H	5.273166	-2.702094	0.397213
H	1.863451	-4.836862	-0.081952
H	-2.387761	-4.445413	-0.722761
H	-2.082069	-2.021188	-0.545871
H	-4.971296	0.181411	-1.922621
H	-7.442797	0.267820	-1.715053
H	-7.082503	0.214843	2.579606
H	-4.610910	0.128026	2.371133
H	-0.326600	8.149496	0.780027
H	-1.302076	6.728027	1.219671
H	0.422081	6.786803	1.647958
H	-1.062026	-6.663049	-1.291699
H	0.574984	-6.838244	-0.644270
H	-0.343918	-6.738611	1.707607
H	-1.988403	-6.556374	1.057523
H	-1.084278	-8.061937	0.773858
H	-0.682409	6.717881	-1.231945
H	1.033256	6.768885	-0.802603

Pt(BPI^{Et})(2)

Pt	0.471886	0.014164	-0.190364
O	-8.324210	-0.247178	0.271668
N	0.445408	2.082248	-0.215604
N	2.818597	2.430296	0.197625
N	2.489604	0.069085	-0.008791
N	2.960606	-2.275843	0.057929
N	0.569304	-2.048299	-0.329998
C	-0.723597	2.713462	-0.480536
H	-1.582500	2.058302	-0.616205
C	-0.870008	4.081630	-0.571047
H	-1.862156	4.479398	-0.786602

C	0.240736	4.918259	-0.382676
C	1.436541	4.274627	-0.113709
H	2.359895	4.832113	0.049818
C	1.552223	2.869167	-0.043873
C	3.218110	1.203020	0.204001
C	4.617453	0.817949	0.470913
C	5.754876	1.570597	0.730915
H	5.710123	2.661045	0.764202
C	6.949450	0.877039	0.947102
H	7.866604	1.433635	1.154411
C	6.992311	-0.522577	0.903202
H	7.942169	-1.033939	1.077120
C	5.841958	-1.273220	0.642060
H	5.864328	-2.364355	0.607406
C	4.660016	-0.576853	0.427319
C	3.285626	-1.028697	0.136237
C	1.720858	-2.775079	-0.200865
C	1.688058	-4.181982	-0.340483
H	2.644081	-4.692088	-0.215506
C	0.528597	-4.876506	-0.630141
C	-0.630970	-4.098776	-0.779955
H	-1.594755	-4.552098	-1.020700
C	-0.564703	-2.731656	-0.625648
H	-1.458667	-2.119448	-0.732683
C	-1.502662	-0.044468	-0.155539
C	-2.729418	-0.081081	-0.059593
C	-4.154869	-0.123173	0.048857
C	-4.976429	-0.161990	-1.098401
H	-4.510380	-0.159524	-2.086350
C	-6.357291	-0.202997	-0.992920
H	-6.989971	-0.233271	-1.882756
C	-6.976411	-0.206385	0.266616
C	-6.179652	-0.168143	1.417605
H	-6.628788	-0.169915	2.411421
C	-4.791586	-0.127389	1.301532
H	-4.181073	-0.098275	2.206817
C	-8.999124	-0.251917	1.502868
H	-8.790093	0.660074	2.088413
H	-10.071500	-0.287746	1.273586
H	-8.735192	-1.133963	2.111662
C	-1.169006	7.047984	-0.160163
H	-1.098806	8.144066	-0.214353
H	-1.957978	6.739040	-0.862235
H	-1.498564	6.781220	0.855863
C	0.498168	-6.373357	-0.740451
H	-0.140500	-6.655350	-1.593512
H	1.511261	-6.740883	-0.965254
C	-0.024004	-7.043543	0.529639
H	0.612494	-6.803382	1.394773
H	-1.047478	-6.713930	0.765412
H	-0.040299	-8.137350	0.412214
C	0.174148	6.417219	-0.493828
H	0.459079	6.684883	-1.527848
H	0.964218	6.844975	0.143616

Pt(BPI^{Et})(3)

Pt	-0.049541	-0.005112	-0.157043
N	-0.187182	-2.071888	-0.205997
N	-2.609067	-2.229231	-0.017821
N	-2.070621	0.101503	-0.078955
N	-2.350137	2.475117	0.068341
N	0.025374	2.062070	-0.254285
C	0.950511	-2.796555	-0.355054
H	1.868934	-2.213119	-0.388009
C	0.991325	-4.169403	-0.459752
H	1.961223	-4.656126	-0.581151
C	-0.200246	-4.910501	-0.416093
C	-1.361343	-4.176193	-0.263466
H	-2.339755	-4.656270	-0.219222
C	-1.366073	-2.765409	-0.161969
C	-2.905102	-0.972747	0.024715
C	-4.282626	-0.480531	0.220796
C	-5.494973	-1.141819	0.366126
H	-5.545282	-2.232230	0.338953
C	-6.637693	-0.357104	0.547323
H	-7.610707	-0.840227	0.665052
C	-6.558551	1.041354	0.581853
H	-7.470901	1.625130	0.726086
C	-5.333623	1.699582	0.435413
H	-5.260504	2.788683	0.461702
C	-4.203830	0.912972	0.255015
C	-2.780919	1.256863	0.071129
C	-1.064293	2.878149	-0.125023
C	-0.910535	4.281841	-0.203128
H	-1.823611	4.865350	-0.078002
C	0.308922	4.886561	-0.443178
C	1.401154	4.018585	-0.607305
H	2.403938	4.396633	-0.816983
C	1.217264	2.657728	-0.506665
H	2.059240	1.977890	-0.623570
C	1.921876	-0.098655	-0.060415
C	3.145159	-0.149794	0.058482
C	0.162296	-7.064449	0.841514
H	0.158597	-8.160701	0.748463
H	1.164795	-6.757175	1.176295
H	-0.553205	-6.787492	1.630326
C	0.470396	6.378204	-0.495773
H	1.112242	6.635759	-1.354348
H	-0.510333	6.842987	-0.680003
C	1.081255	6.947708	0.783559
H	0.443928	6.735808	1.655374
H	2.073764	6.514418	0.981189
H	1.198923	8.038690	0.704654
C	-0.205143	-6.409987	-0.489301
H	0.509111	-6.730771	-1.264873
H	-1.200482	-6.753399	-0.810232
C	4.566765	-0.214176	0.154389
C	5.282508	-0.072986	1.331528
C	5.444329	-0.434121	-0.964879

H	4.881866	0.100751	2.329702
H	5.086213	-0.569344	-1.986675
C	6.764934	-0.453103	-0.610609
H	7.633038	-0.598769	-1.253007
S	6.971385	-0.205175	1.078330

Pt(BPI^{Et})(4)

C	-5.003534	-0.288245	1.469012
C	-4.388082	-0.201591	0.204271
C	-5.215548	-0.187964	-0.936360
C	-6.600081	-0.258261	-0.822435
C	-7.168631	-0.343151	0.442936
C	-6.387124	-0.358979	1.592254
C	-2.965172	-0.131507	0.080775
C	-1.732516	-0.074161	-0.035084
Pt	0.238259	0.014386	-0.132904
N	0.361411	-2.049766	-0.263642
C	1.531324	-2.756298	-0.202457
C	1.515447	-4.164304	-0.333829
C	0.352774	-4.881922	-0.542185
C	-0.828246	-4.126162	-0.616521
C	-0.776617	-2.756933	-0.476675
N	2.775537	-2.235670	-0.020124
C	3.082732	-0.983447	0.043436
N	2.260811	0.100873	-0.051274
C	2.982956	1.246400	0.118235
C	4.401182	0.884183	0.304088
C	4.463865	-0.509449	0.255189
C	5.667227	-1.186891	0.400063
C	6.818280	-0.417900	0.596549
C	6.755279	0.980813	0.645930
C	5.539271	1.655152	0.499888
N	2.565309	2.467294	0.132513
C	1.281232	2.886674	-0.038185
N	0.178962	2.083063	-0.153024
C	-1.012089	2.698800	-0.347682
C	-1.183876	4.064749	-0.422851
C	-0.076928	4.917551	-0.293937
C	1.141120	4.290579	-0.095635
C	-0.172358	6.415635	-0.394121
C	-1.500845	7.024937	0.027322
C	0.343098	-6.379607	-0.642654
C	-0.056082	-7.051078	0.670789
F	-8.497771	-0.410969	0.557384
H	-1.868376	2.033263	-0.439169
H	-2.192571	4.448149	-0.579789
H	2.064019	4.860857	0.018940
H	5.479148	2.744730	0.537886
H	7.673640	1.552031	0.801740
H	7.784748	-0.913859	0.714709
H	5.704825	-2.277470	0.361295
H	2.487028	-4.655875	-0.269039
H	-1.797716	-4.598623	-0.787694
H	-1.687698	-2.163705	-0.529426

H	-4.757574	-0.121480	-1.925419
H	-7.245411	-0.248257	-1.703161
H	-6.869193	-0.426394	2.569636
H	-4.379436	-0.300237	2.364860
H	-1.450443	8.122114	-0.026718
H	-2.328399	6.706446	-0.624223
H	-1.760764	6.750268	1.061343
H	-0.359553	-6.679675	-1.437034
H	1.340969	-6.728398	-0.950287
H	0.647273	-6.793470	1.477126
H	-1.061759	-6.738724	0.991600
H	-0.061512	-8.145744	0.560647
H	0.041577	6.691261	-1.443077
H	0.650322	6.853148	0.193406

Pt(BPI^{Et})(5)

C	1.752561	4.239636	-0.084557
C	1.833618	2.827451	-0.076144
N	0.691414	2.074131	-0.066703
C	-0.487745	2.745899	-0.062198
C	-0.603843	4.118261	-0.068025
C	0.549355	4.919042	-0.082321
N	3.109833	2.354911	-0.079667
C	3.472143	1.116120	-0.078728
N	2.688561	0.000129	-0.075956
C	3.472262	-1.115772	-0.080410
C	4.887345	-0.697649	-0.083617
C	4.887271	0.698151	-0.082551
C	6.071252	-1.423071	-0.087452
C	7.267989	-0.700135	-0.089919
C	7.267915	0.700897	-0.088842
C	6.071101	1.423702	-0.085275
N	3.110079	-2.354598	-0.083189
C	1.833921	-2.827284	-0.080235
N	0.691634	-2.074115	-0.069495
C	-0.487453	-2.746027	-0.065759
C	-0.603395	-4.118387	-0.073612
C	0.549900	-4.919016	-0.089351
C	1.753021	-4.239469	-0.090794
Pt	0.661891	-0.000001	-0.066900
C	-1.316238	-0.000112	-0.060941
C	-2.547601	-0.000133	-0.061252
C	-3.977393	-0.000047	-0.064965
C	-4.720115	-0.028478	1.130973
C	-6.107593	-0.028733	1.133846
C	-6.844181	0.000134	-0.074422
C	-6.099636	0.028951	-1.277833
C	-4.712297	0.028518	-1.265790
C	0.475197	-6.418181	-0.068156
C	0.172648	-6.972868	1.323046
C	0.474483	6.418164	-0.058827
C	0.172797	6.970689	1.333426
N	-8.216573	0.000228	-0.079095
H	-1.376144	2.116370	-0.057173

H	-1.603726	4.557346	-0.068542
H	2.707380	4.766926	-0.097462
H	6.059697	2.515549	-0.084583
H	8.220984	1.235362	-0.090884
H	8.221116	-1.234494	-0.092786
H	6.059961	-2.514919	-0.088433
H	2.707897	-4.766630	-0.104659
H	-1.603228	-4.557586	-0.074632
H	-1.375919	-2.116602	-0.059688
H	-4.173036	0.051024	-2.215969
H	-6.611563	0.052169	-2.239928
H	-6.625903	-0.051797	2.092527
H	-4.187199	-0.051012	2.084723
H	0.115985	8.069214	1.310492
H	-0.786955	6.591280	1.716676
H	0.956271	6.685332	2.051792
H	-0.308203	-6.744980	-0.771509
H	1.426442	-6.833066	-0.435612
H	0.955641	-6.688566	2.042355
H	-0.787378	-6.594127	1.706272
H	0.115927	-8.071360	1.298382
H	-0.309426	6.745941	-0.761149
H	1.425437	6.833725	-0.426276
C	-8.941306	-0.027740	1.165229
C	-8.932718	0.027640	-1.328393
H	-10.018251	-0.019816	0.958693
H	-8.720549	-0.934009	1.758169
H	-8.715485	0.848049	1.800406
H	-10.011050	0.019515	-1.129275
H	-8.708124	0.933787	-1.920081
H	-8.702318	-0.848257	-1.961790
